# Metabolic flux-driven sialylation alters internalization, recycling, and drug sensitivity of the epidermal growth factor receptor (EGFR) in SW1990 pancreatic cancer cells

**DOI:** 10.18632/oncotarget.11582

**Published:** 2016-08-24

**Authors:** Mohit P. Mathew, Elaine Tan, Christopher T. Saeui, Patawut Bovonratwet, Samuel Sklar, Rahul Bhattacharya, Kevin J. Yarema

**Affiliations:** ^1^ Department of Biomedical Engineering and the Translational Tissue Engineering Center, The Johns Hopkins University, Baltimore, Maryland, USA; ^2^ Current address: Yale School of Medicine, New Haven, Connecticut, USA

**Keywords:** pancreatic cancer, sialic acid, galectin, epidermal growth factor receptor (EGFR), glycobiology

## Abstract

In prior work we reported that advanced stage, drug-resistant pancreatic cancer cells (the SW1990 line) can be sensitized to the EGFR-targeting tyrosine kinase inhibitors (TKIs) erlotinib and gefitinib by treatment with 1,3,4-*O*-Bu_3_ManNAc (*Bioorg. Med. Chem. Lett.* (2015) 25(6):1223-7). Here we provide mechanistic insights into how this compound inhibits EGFR activity and provides synergy with TKI drugs. First, we showed that the sialylation of the EGFR receptor was at most only modestly enhanced (by ∼20 to 30%) compared to overall ∼2-fold increase in cell surface levels of this sugar. Second, flux-driven sialylation did not alter EGFR dimerization as has been reported for cancer cell lines that experience increased sialylation due to spontaneous mutations. Instead, we present evidence that 1,3,4-*O*-Bu_3_ManNAc treatment weakens the galectin lattice, increases the internalization of EGFR, and shifts endosomal trafficking towards non-clathrin mediated (NCM) endocytosis. Finally, by evaluating downstream targets of EGFR signaling, we linked synergy between 1,3,4-*O*-Bu_3_ManNAc and existing TKI drugs to a shift from clathrin-coated endocytosis (which allows EGFR signaling to continue after internalization) towards NCM endocytosis, which targets internalized moieties for degradation and thereby rapidly diminishes signaling.

## INTRODUCTION

In mammals, glycosylation is a ubiquitous co/post-translational modification of proteins and lipids that modulates the activities of these molecules in many ways that – despite decades of study – often remain poorly understood. An illustration of a recent, unexpected glycosylation-based result is the ability of 1,3,4-*O*-Bu_3_ManNAc to sensitize drug-resistant pancreatic cancer cells to tyrosine kinase inhibitors (TKIs) [1]. To elaborate briefly, this compound is a “high flux” *N*-acetylmannosamine (ManNAc) analog that increases sialylation [2–5]; consequently it is counterintuitive that such a compound could have anti-cancer potential because sialic acid has generally been regarded as cancer-promoting. This sugar occurs in many tumor-associated carbohydrate antigens (TACAs) such as the sialylated Tn antigen (sTn), sialyl Lewis X (sLe^X^) and ganglioside GM3 [6, 7] and its bulk chemical properties – for example when it is assembled into polysialic acid – can be anti-adhesive and provide a mechanism for cancer cells to detach from a primary tumor to initiate metastasis.

Links between sialic acid and oncogenesis suggests that increased sialylation would be counter-productive in cancer therapy thereby posing a conundrum for exploiting otherwise promising metabolic oligosaccharide engineering strategies to treat cancer. Metabolic oligosaccharide engineering (MOE [8, 9], also known as metabolic glycoengineering, MG or MGE [10]) refers to a method where non-natural monosaccharides are supplied to living cells or animals to modulate glycosylation (detailed background information is provided elsewhere [11–14]). To illustrate this conundrum, MOE can be used to install non-natural sialic acids in TACAs for delivery of diagnostic [15, 16] and therapeutic [17] agents or even for fluorinated sugars intended to have anti-metastatic properties [9,18,19]. At the same time, however, these approaches could promote cancer progression by increasing overall sialylation and, for this reason (and others beyond the scope of this discussion), MOE has made only halting progress towards clinical adoption.

Intriguingly, several reports provide a counterpoint to the widely held assumption that sialylation is synonymous with increased carcinogenicity. For example, our group found that 1,3,4-*O*-Bu_3_ManNAc – a “pro-drug” that is activated by intracellular esterases to generate ManNAc [20] – promotes high levels of flux through the sialic acid biosynthetic pathway [3] and can double cell surface sialylation in human cancer cells [4]. Surprisingly however, this compound had only a modest (in fact almost negligible) impact on endpoints related to metastasis such as cell motility [4]. One explanation for this muted response to increased sialylation is that once cancer cells have attained a disease-promoting level of this sugar, any additional increase may not be capable of further exacerbating cancer progression. Indeed, although sialylation is associated with many aspects of oncogenesis [21, 22], too large of an increase may actually be detrimental. This idea is consistent with descriptions of only “slightly increased” levels of sialic acid in some types of cancer [23] and feedback mechanisms that carefully titer metabolic flux (i.e., generation of ManNAc from UDP-GlcNAc) into the sialic acid biosynthetic pathway [24,25].

The epidermal growth factor receptor (EGFR) is an oncogenic protein linked to poor prognosis in pancreatic (and other) cancers [26–28] that illustrates how hypersialylation can deter cancer progression. For context, the overall glycosylation status of EGFR has been linked to changes in receptor activity as well as to overall cell behavior [29, 30]. For example, genetic deletion of the Asn-420 glycosylation site enables ligand-free activation of EGFR [31] and inhibition of N-glycosylation with tunicamycin sensitizes human non-small cell lung cancer cells to erlotinib [32]. Focusing on sialic acid, increased levels of this sugar observed in certain cancer cell lines were found several years ago to inhibit EGFR activity in lung cancer cells [29]. In subsequent work, the impact of sialylation on EGFR activity was through the over-expression of sialyltransferases [33], which diminished EGFR activity and through sialidase treatment, which removed sialic acid and promoted EGFR signaling [34].

Building on these findings, our group recently showed that a “small molecule” approach using 1,3,4-*O*-Bu_3_ManNAc could reproduce drug sensitization achieved by genetic manipulation of sialylation. Looking forward to clinical translation, our approach is important because we rely on conventional drug strategies rather than gene therapy approaches that have yet to be validated for use in human patients and furthermore, our approach is transient and reversible and thus can avoid long-term harm to healthy tissues (e.g., increased sialylation that can promote tumorogenesis over a period of months or years can be avoided by our strategy). In particular, 1,3,4-*O*-Bu_3_ManNAc sensitized drug-resistant pancreatic cancer cells to the tyrosine kinase inhibitors (TKIs) erlotinib and gefitinib [1] that are currently used as cancer therapeutics but are only modestly effective because of rapid onset drug resistance in patients [35]. Based on the clinical promise of counteracting drug resistance, especially in difficult-to-treat malignancies such as pancreatic cancer, we investigated the impact of 1,3,4-*O*-Bu_3_ManNAc on EGFR signaling to gain insight into the underlying molecular mechanisms by which this compound attenuated oncogenic signaling and to understand the unusual synergy between TKIs and increased cellular sialylation supported by this compound.

## RESULTS

### 1,3,4-*O*-Bu_3_ManNAc treatment has a minor impact on EGFR sialylation

**Figure 1:**
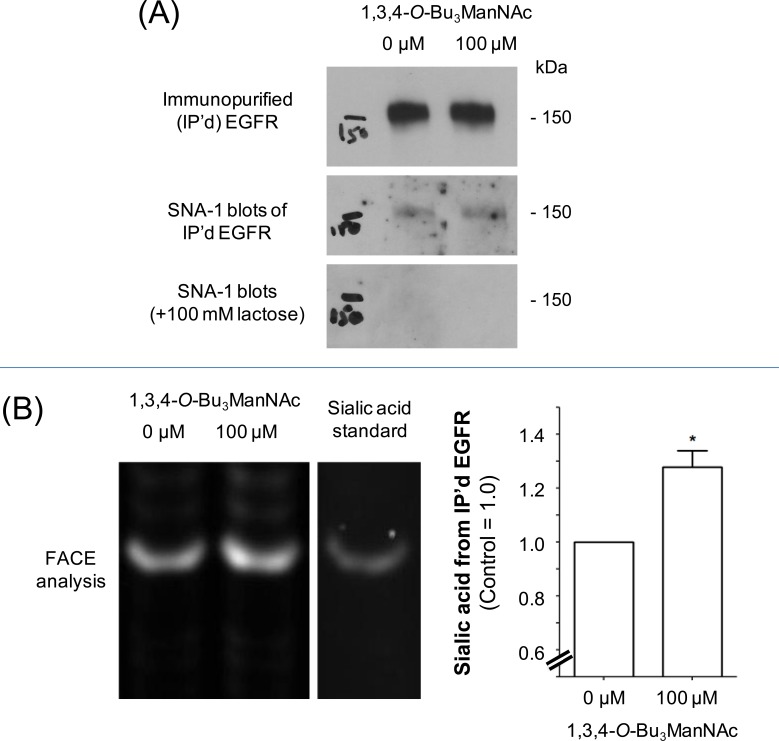
Sialylation of immunopurified (IP'd) EGFR from SW1990 cells Cells were treated with 100 μM (or 0 μM for controls) 1,3,4-*O*-Bu_3_ManNAc and EGFR was IP'd from each condition. (**A**) One aliquot of the IP'd EGFR was separated using PAGE and the resulting blots were probed with an EGFR-recognizing antibody or HRP-linked SNA-1 lectin; the SNA-1 staining was ablated by the binding competitor lactose. The data from the western blot was quantified and normalized to EGFR levels as shown in the the bar graph, which verifies that there was a small increase in α2,6 sialylation of EGFR in the 1,3,4-*O*-Bu_3_ManNAc-treated cells. (**B**) A second aliquot of IP'd EGFR was digested with sialidase, which was analyzed by Fluorescent Assisted Carbohydrate Electrophoresis (FACE). Quantification of the FACE bands (normalized to EGFR levels determined from the western blots) provided independent verification that overall sialylation of EGFR increased with 1,3,4-*O*-Bu_3_ManNAc treatment Each experiment includes at least three biological replicates and the bands were quantified using Image J with data expressed as mean ± standard error mean (SEM). * indicates a *p* value of < 0.05.

In previous studies we characterized the glycosylation of SW1990 cells by using “glycosite” glycoproteomic analysis (4) as well as through N-glycan profiling [5]. As outlined in detail in the Supplemental Material, 1,3,4-*O*-Bu_3_ManNAc treatment increased the sialylation of one N-glycan site on EGFR (overall, EGFR has 11 sequons for N-glycan attachment with eight of these sites typically occupied [36]). To quickly summarize this prior work here in the main document, it suggested that 1,3,4-*O*-Bu_3_ManNAc had only a minor impact on EGFR compared to its much higher enhancement of overall cell surface sialylation. To confirm this premise in the current study, we used two additional methods to measure EGFR sialylation. In both cases we began by immunopurifying EGFR from control and 1,3,4-*O*-Bu_3_ManNAc-treated cells. In the first set of experiments, the purified EGFR was quantified by using western blots (Figure 1A) and in parallel stained for α2,6 sialic acid using HRP-linked SNA-1 lectin. Examination of the lectin blots indicated a slight trend towards increased sialylation upon 1,3,4-*O*-Bu_3_ManNAc treatment but rigorous quantification was not possible due to artifacts in the blots. In an independent method, the immunopurified EGFR was incubated with sialidase and the released sialic acid was quantified using FACE analysis (Figure 1B). These experiments showed an increase of ∼20 to 30% in EGFR sialylation in SW1990 cells, which was consistent with the previously-reported minimal increase in this endpoint [4] and markedly lower than the ∼2-fold increase in global surface sialylation in cells treated with 1,3,4-*O*-Bu_3_ManNAc under identical conditions.

Our finding that 1,3,4-*O*-Bu_3_ManNAc had a only a minor impact on EGFR sialylation differed from reports where five of EGFR's N-glycan sites experienced a two-fold or higher increase in sialic acid [29]. A comparison of these studies suggested that genetic manipulation compared to our flux-based method resulted in clearcut differences to EGFR sialylation; in particular, our approach had a disproportionately minor effect on EGFR compared to overall changes to cell surface sialylation. Despite this disparity, a critical endpoint of increased sialylation achieved through 1,3,4-O-Bu_3_ManNAc – i.e., diminution of EGFR signaling and sensitization to TKI drugs – was remarkably similar to genetically modified cells [34]. However, as described in this report, although the ultimate outcome of each approach converged on the same endpoint of diminishing EGFR signaling and sensitizing cells to TKI drugs, metabolic flux-based changes to EGFR function through a different mechanism that affects receptor activity and trafficking in ways that supersede (but potentially complement) the previously-reported mechanism where increased sialylation inhibits EGFR signaling by decreasing receptor dimerization [29, 33, 34].

### FRAP assays show minimal changes in receptor-ligand binding affinity, thereby discounting the role of dimerization

We tested whether changes to sialylation achieved through 1,3,4-*O*-Bu_3_ManNAc treatment had a similar effect on EGFR dimerization as discussed above; based on the minimal impact of 1,3,4-*O*-Bu_3_ManNAc on EGFR sialylation (Figure 1), we did not anticipate that this would be the case but wanted to experimental confirm this premise. This expectation was supported by western blot assays conducted following the procedures described by Liu *et al*., [29] that were not able to reproduce the published results where increased sialylation inhibited EGFR dimerization. Instead, although we did observe a slight trend towards reduced dimerization, we were not able to achieve a statistically significant result despite repeating the experiment multiple times.

To confirm that 1,3,4-*O*-Bu_3_ManNAc had minimal (if any) impact on EGFR dimerization, we explored additional methods to assess this endpoint. In the first of these experiments, we used fluorescent recovery after photobleaching (FRAP) assays to evaluate receptor (EGFR)-ligand (EGF) binding kinetics, which are influenced by receptor dimerization [37–39]. A portion of the cell membrane was photobleached while the cells were in a bath containing Alexa Fluor 488-conjugated EGF. The rate at which bleached EGF molecules were released and unbleached Alexa Fluor 488-conjugated EGF from solution bound to the vacated receptors was measured by monitoring the recovery of fluorescence. The t_0.5_ value is inversely proportional to the rate at which bleached EGF molecules are released and unbleached Alexa Fluor 488-conjugated EGF from solution bind to the vacated receptors, thereby providing a quantitative measurement of receptor-ligand binding.

**Figure 2:**
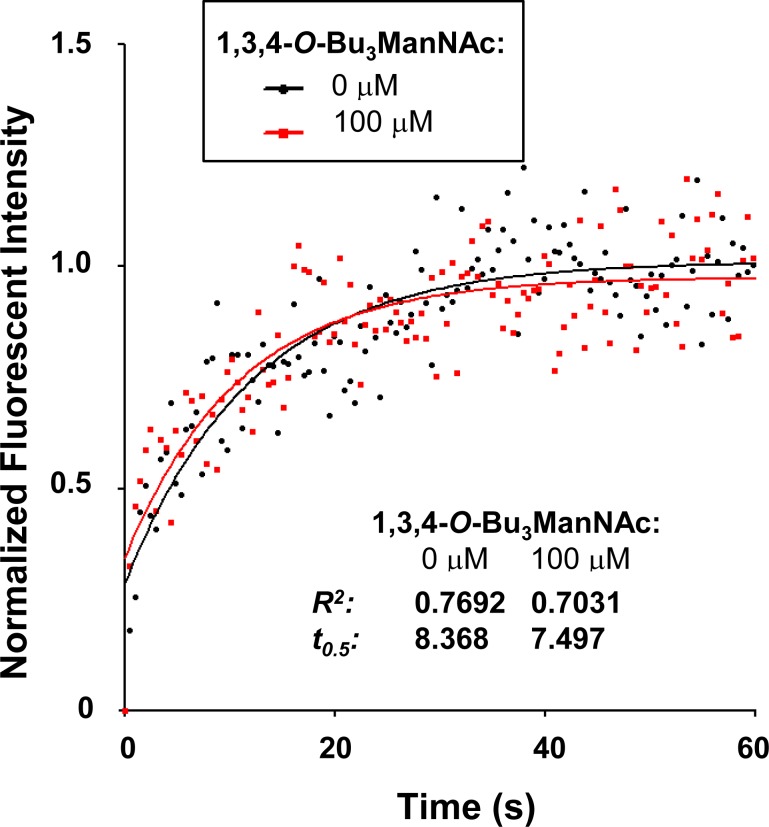
EGFR Ligand affinity measured using a FRAP assay A representative fluorescent recovery after photobleaching (FRAP) assay is shown, in which recovery rates are inversely proportional to t_0.5_ when Alexafluor 488-conjugated EGF is present in the bath. This experiment indicated that receptor ligand affinities were not measurably different in the presence or absence of 1,3,4-*O*-Bu_3_ManNAc.

Because ligand binding is linked to the dimerization status of EGFR [37–39], EGF binding kinetics would be expected to differ between 1,3,4-*O*-Bu_3_ManNAc-treated and control cells if EGFR dimerization is perturbed by analog treatment. The t_0.5_ values determined for 1,3,4-*O*-Bu_3_ManNAc-treated and control cells, however, were statistically identical (7.497s and 8.368s, respectively, Figure 2). This experiment provided added evidence that a flux-based increase in sialylation modulates EGFR activity through a mechanism different than previously reported changes to receptor dimerization.

### Surface localization of EGFR was decreased by 1,3,4-*O*-Bu_3_ManNAc treatment, supporting a galectin lattice mechanism

**Figure 3:**
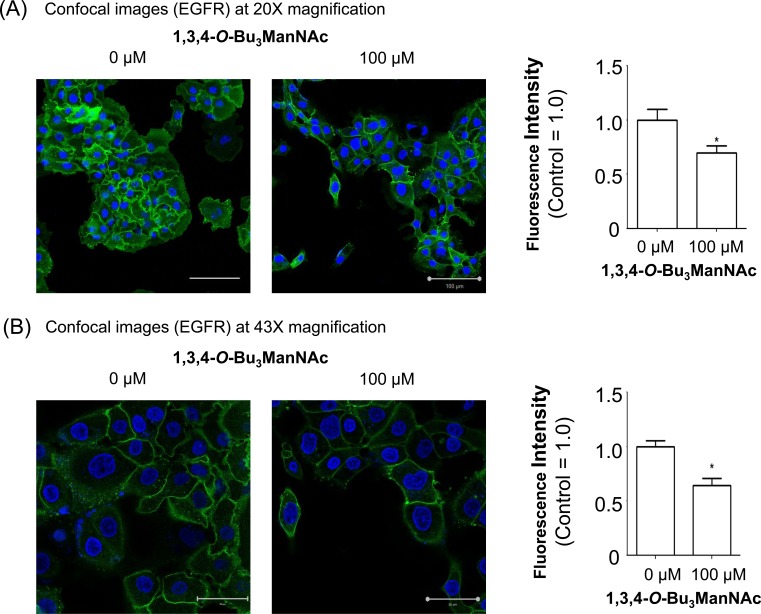
Confocal imaging of EGFR in SW1990 cells treated with 1,3,4-*O*-Bu_**3**_ManNAc The treated cells, in comparison with untreated controls, were imaged at (**A**) 20X and (**B**) 43X magnifications after being fixed and stained with Alexa Fluor-488 conjugated EGFR mAb (green) and DAPI (blue). Quantification of mAb staining, normalized to DAPI, showed a decrease in surface localized EGFR when SW1990 cells were treated with 100 mM 1,3,4-***O***-Bu_3_ManNAc as shown in the bar graphs. Each experiment includes at least three biological replicates and the fluorescence intensity of Alexa Fluor-488 and DAPI were quantified using Image J with the resulting data expressed as the mean value +/− standard error of the mean (SEM). Asterisks (*) indicate a p value of < 0.05. Scale bars represent 100 μm and 50 μm, respectively for Panels A and B.

Based on the evidence that 1,3,4-*O*-Bu_3_ManNAc had minimal impact on EGFR dimerization, we reasoned that this compound's ability to reduce EGFR signaling involved a different (or additional) mechanism. One hint from the saturation binding assays we previously reported [1] was that the binding of EGF to the cell surface was lower in 1,3,4-*O*-Bu_3_ManNAc-treated compared to control cells. The earlier study, however, did not distinguish between two explanations for this finding: first, changes to ligand affinity due to factors such as EGFR dimerization or second, reduced surface localization of the receptor. In the current study having cast doubt on a major role for changes to ligand affinity by not gaining evidence for the dimerization hypothesis we pursued the second possibility‵, which was reduced surface display of EGFR. To monitor this parameter‵, we directly labeled surface EGFR using an Alexa Fluor-488-conjugated mAb followed by analysis using confocal microscopy (Figure 3). In this experiment surface EGFR was measured using the selective permeabilization method previously reported for EGFR (40,41) and other surface receptors [41,42]. In particular, we followed the procedure described by Mardones and coworkers who showed that ectodomain targeting EGFR antibodies of the type we used only stain cell surface-localized EGFR when the cells are fixed without permeabilization [41]. Quantification of the resulting fluorescence at two magnifications (20x, panel A and 43x, panel B) confirmed that analog treatment resulted in decreased display of cell surface localized EGFR. The images presented in Figure 3 show some heterogeneity in EGFR expression, consistent with the existence of “side populations” in pancreatic cancer cell lines (as described by Yao *et al*., [43]); in the current publication – which aims to describe a new mechanism for modulating EGFR activity *via* an MOE approach – this nuance in cell to cell variability is less important than our goal of describing the overall effects of increased flux-based sialylation on the trafficking of EGFR in cells that express this oncogenic protein.

### Mathematical modeling and experimental evidence suggests that the galectin lattice modulates EGFR activity in 1,3,4-*O*-Bu_3_ManNAc-treated cells

**Figure 4:**
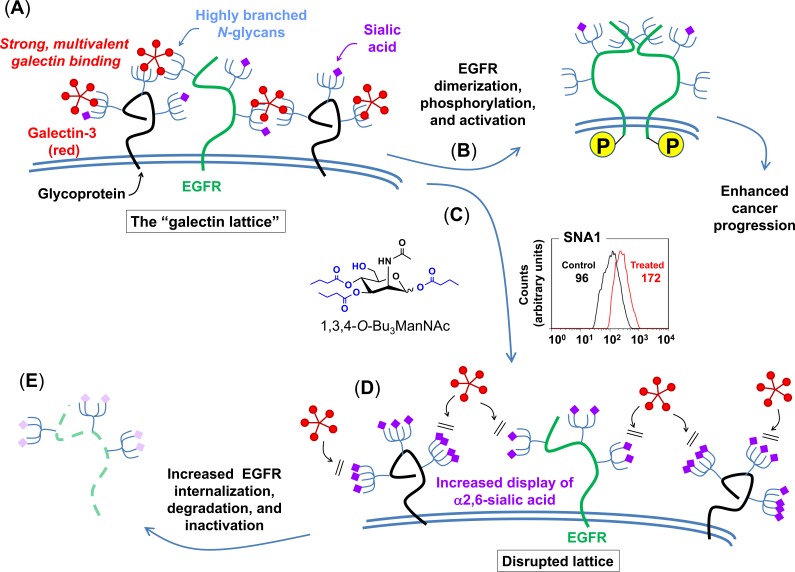
Proposed galectin lattice-mediated mechanism for modulation of EGFR signaling through 1,3,4-*O*-Bu_3_ManNAc treatment The combined modeling and early experimental results were consistent with the depicted biochemical mechanism where destabilization of the galectin lattice occurs due to the masking of galectin-binding epitopes by analog-driven increased sialylation of *N*-linked glycans. (**A**) Cancer cells often have highly organized, less-mobile surface receptors (e.g., the green structure represents EGFR while the black structures represent any other cell surface glycoprotein) in part because of a highly formed galectin lattice. (**B**) One result of a strong galectin lattice is lengthened residence times for EGFR on the cell surface [45], resulting in enhanced phosphorylation that lead to increased downstream EGFR signaling, which contributes to cancer progression. (**C**) 1,3,4-*O*-Bu_3_ManNAc treatment leads to an increase in sialylation of *N*-linked glycans bound to EGFR, based on lectin-staining data (specifically SNA binding as shown in the representative FACS plot depicts globally increased expression of α2,6-linked sialic upon 1,3,4-*O*-Bu_3_ManNAc treatment, which is consistent with both the data shown in Figure 1 and our previous results [4]); glycans terminated with α2,6-linked sialic acids mask galactose residues and negatively regulate galectin binding [48]. (**D**) In turn, reduced galectin binding decreases lattice strength, thereby increasing the surface mobility of EGFR and enhancing its removal from the cell surface [45]. (**E**) Ultimately – over time periods longer (e.g., 30 to 90 min) than the 2 min time frame investigated in our previous work (1) – this increased rate of internalization predicts faster inactivation of EGFR, which is computationally and experimentally demonstrated subsequently in this report.

To generate hypotheses to describe how 1,3,4-*O*-Bu_3_ManNAc could modulate EGFR activity through a non-dimerization based mechanism, western blot and saturation binding results from our previous work [1] and the surface localization assays reported herein (e.g., in Figure 3) were analyzed by using a macroscopic cell-level model of EGFR trafficking [44]. This model, which contains five basic components (ligand binding, synthesis, internalization, degradation, and recycling) that are in most cases subdivided into additional steps, is described in detail in the Supplemental Material (e.g., in Figure S2). This model was implemented to explore the impact of 1,3,4-*O*-Bu_3_ManNAc on EGFR trafficking by analyzing each parameter on its own; none of these model simulations were consistent with our experimental data. We then simulated the parameters in pairwise combinations, which resulted in a single “hit” where the modeled result was consistent with experimental data. Specifically, the results shown in Figure S5, Panel (A) – which were obtained by simultaneously varying the simulated internalization and recycling rates – provided a match between modeled and experimental results. These simulations predicted (as shown in the upper graph) that an increase in the internalization rate (k_e_) along with a decrease in the recycling rate (1/k_x_) would result in decreased EGFR phosphorylation accompanied by a slight increase in overall EGFR levels. Significantly, this set of variables also predicted (lower graph) the lower initial EGFR levels observed on the cell surface after treatment with 1,3,4-*O*-Bu_3_ManNAc (Figure 3) observed before the simulated addition of EGF. A survey of the literature provided a biochemical mechanism consistent with the modeled simulations insofar as sialylation can disrupt the galectin lattice, which in turn can influence EGFR activity in cancer [45] as depicted in Figure 4 and described in more detail in the Discussion, below. To gain support for this mechanism, we next conducted experiments to verify that a 1,3,4-*O*-Bu_3_ManNAc-driven increase in sialylation could disrupt the galectin lattice, modulate EGFR trafficking, and ultimately attenuate downstream signaling.

### 1,3,4-*O*-Bu_3_ManNAc increases membrane fluidity

**Figure 5:**
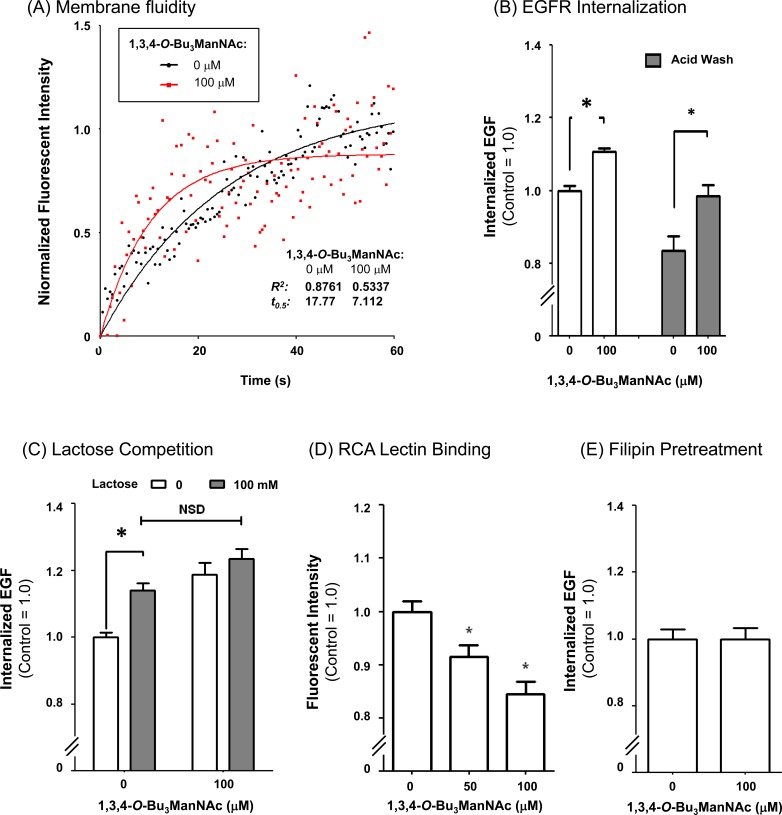
EGFR internalization assays (**A**) FRAP assays, conducted in the absence of excess fluorescently-labeled EGF, indicated that the recovery rate (*t*) was over twice as fast in cells treated with 1,3,4-*O*-Bu_3_ManNAc indicated greater membrane fluidity consistent with decreased galectin lattice strength (as outlined in Figure 3). (**B**) Internalization assays conducted in the presence of fluorescently-labeled EGF for 30 min at 37 C showed that 1,3,4-*O*-Bu_3_ManNAc treatment led to a significant increase in EGF, and by extension EGFR, internalization. (**C**) Lactose pretreatment, a competitive inhibitor of galectin binding, led to an increase in internalization in control cells comparable to the increase caused by 1,3,4-*O*-Bu_3_ManNAc. (**D**) RCA lectin binding decreased significantly on treatment with 1,3,4-*O*-Bu_3_ManNAc. (**E**) Internalization measured after filipin pretreatment was not significantly different between the control and treated samples. At least three biological replicates were carried out for each experiment with data expressed as mean ± standard error mean (SEM) and * indicates *p* < 0.05.

To evaluate whether increased membrane fluidity predicted from the hypothesized attenuation of the galectin lattice contributed to observed increased internalization of EGFR, FRAP assays were conducted in the absence of unbound Alexa Fluor 488-conjugated EGF. In this experiment, which differs from the results shown in Figure 2 where the bath contained an excess of fluorescently-labeled EGF, membrane fluidity was monitored by measuring the rate that fluorescently-labeled EGF already bound to receptors adjacent to the bleached region diffuses into the bleached areas. The t_0.5_ values determined in this experiment, which are inversely proportional to the rate of diffusion were noticeably different in the 1,3,4-*O*-Bu_3_ManNAc treated cells that had faster diffusion rates compared to non-treated controls (control t_0.5_= 17.77, treated t_0.5_= 7.112, Figure 5A). The observed trends were repeatable across multiple experiments and supported the premise that 1,3,4-*O*-Bu_3_ManNAc modulated cell surface trafficking dynamics. To further confirm this result, an independent flow cytometry assay showed a similar increase in membrane fluidity and subsequent changes in EGFR internalization as were observed in the FRAP assays (Figure 5B). Next, lactose (a galectin binding inhibitor [46]) was used to competitively inhibit galectin binding to 1,3,4-*O*-Bu_3_ManNAc-treated cells to further confirm the role of the galectin lattice mechanism in EGFR internalization. The presence of lactose increased the internalization rate of EGFR in untreated control cells to levels statistically identical to those observed in analog-treated cells (Figure 5C). This result showed that the inhibitory effects of increased sialylation, which attenuates lattice strength by blocking galectin binding to surface receptors, can be mimicked by lactose competition that also disrupts the binding of these lectins.

Finally, a binding assay using *Ricinus communus* agglutinin (RCA), a lectin that recognizes terminal galactose residues (which are the critical binding epitopes for galectins when they are presented on highly-branched *N*-glycans) showed decreased signal in 1,3,4-*O*-Bu_3_ManNAc-treated cells (Figure 5D). The affinity of RCA and galectins for terminal galactose residues is regulated in a yin-yang manner by sialylation because sialic acids mask binding sites for these lectins [47]. The RCA results (both from this study (Figure 5D) and from our previous investigation of the SW1990 pancreatic cancer cell line, [4]) therefore indicated that potential galectin binding sites were masked by increased levels of sialylation in 1,3,4-*O*-Bu_3_ManNAc-treated cells. In particular, α2,6-linked sialic acids that block galectin binding (48) approximately double in 1,3,4-*O*-Bu_3_ManNAc-treated SW1990 cells based on *Sambucus nigra* agglutinin (SNA) staining [4, 5].

### 1,3,4-*O*-Bu_3_ManNAc shifts internalization towards NCM

Beyond providing clues that 1,3,4-*O*-Bu_3_ManNAc treatment increased the rate of EGFR internalization consistent with the galectin lattice mechanism outlined in Figure 4, which we experimentally confirmed as just described, the mathematical modeling presented in the Supplemental Material predicted a decreased recycling rate (1/k_x_). A biochemical mechanism consistent with this prediction was a shift towards non-clathrin mediated (NCM) endocytosis and away from clathrin mediated internalization because – unlike clathrin mediated internalization where signaling can continue and indeed be amplified – NCM-internalized moieties are directed for rapid degradation rather than recycling [49]. This insight led us to investigate NCM endocytosis in more detail by treating cells with filipin, which is an inhibitor of non-chathrin mediated (NCM) endocytosis. As shown in Figure 5E, filipin ablated the 1,3,4-*O*-Bu_3_ManNAc-driven increase in EGFR internalization, indicating that this mode of internalization played an important role in EGFR trafficking in analog-treated cells compared to untreated controls where clathrin-coated internalization played a dominant role in receptor trafficking.

**Figure 6:**
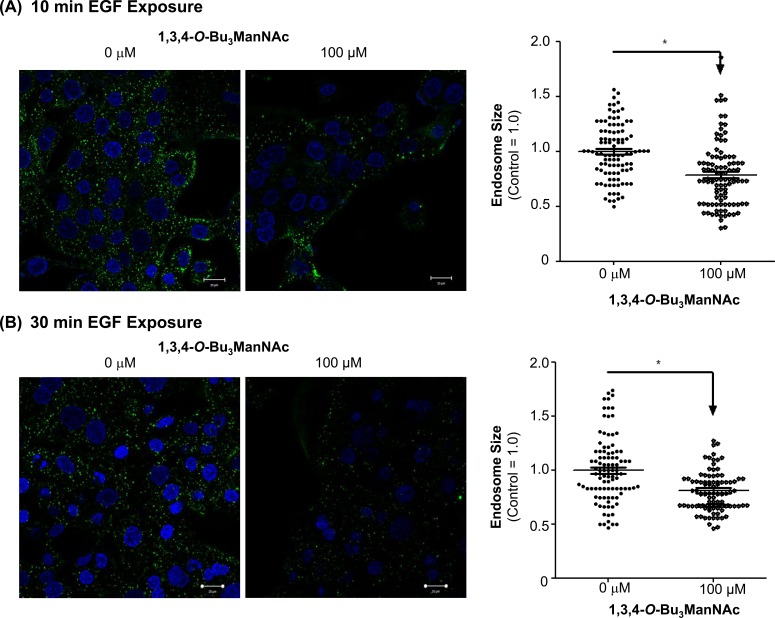
Confocal images of SW1990 cells after (**A**) 10 min and (**B**) 30 min of exposure to 2 μg/ml Alexafluor 488-conjugated EGF at 37oC showed a significantly greater density of larger endosomes in non-treated controls compared to cells treated with 1,3,4-*O*-Bu_3_ManNAc. The endosomes were sized using ImageJ and * indicates a p value of < 0.05. Scale bars represent 20 μm.

To further confirm that NCM endocytosis played an important role in EGFR internalization in 1,3,4-*O*-Bu_3_ManNAc treated cells, we monitored time points longer than the two minute intervals used in our initial experiments (and modeling simulations) because NCM endocytosis requires 30 to 90 min to fully route cell surface elements towards degradation [49]. We reasoned that there should be evidence of a shift from clathrin-coated internalization to NCM endocytosis before full degradation takes place, therefore we assessed these two modes of internalisation at 10 and 30 min time points that precede the onset of degradation by measuring endosome size by visualizing endocytosis; this parameter was chosen for analysis because clathrin-coated endosomes are larger (∼100-150 nm) [50–52] than NCM endosomes (∼50-80 nm) [53, 54]. In these experiments, 1,3,4-*O*-Bu_3_ManNAc-treated and control cells were incubated with Alexa Fluor 488-conjugated EGF for 10 min (Figure 6A) or 30 min (Figure 6B) at 37°C, fixed, and then imaged using confocal microscopy. Endosome sizing by Image J showed a significant shift in endosome population from larger endosomes toward smaller endosomes in the analog-treated cells compared to untreated controls. This shift toward smaller endosomes was consistent with increased NCM endocytosis at the expense of clathrin-coated endocytosis and provided additional support for the hypothesis that EGFR internalization became biased towards NCM endocytosis upon 1,3,4-*O*-Bu_3_ManNAc treatment.

**Figure 7:**
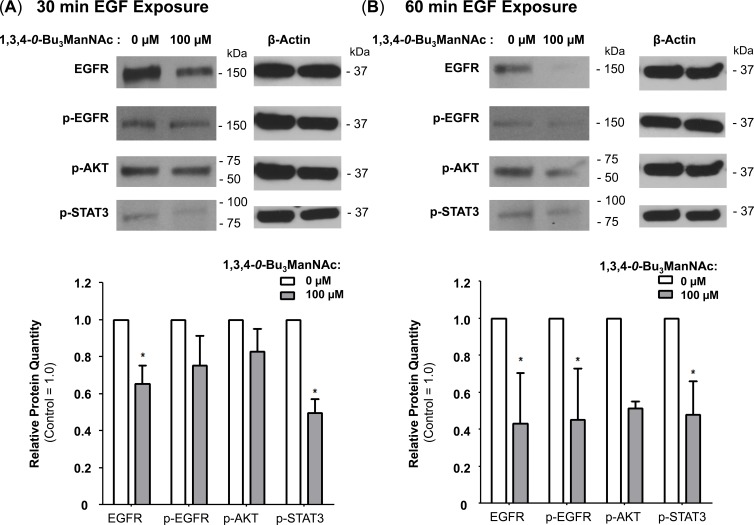
EGFR degradation is enhanced by 1,3,4-*O*-Bu_3_ManNAc Western blot analysis of SW1990 cells exposed to 10 ng/mL of EGF for (**A**) 30 min or (**B**) 60 min after incubation with (or without) 1,3,4-*O*-Bu_3_ManNAc revealed that longer exposures to EGF led to decreased EGFR levels for the treated cells. At least 3 biological replicates were carried out for each experiment with data expressed as mean ± standard error mean (SEM). * indicates a p value of < 0.05.

Finally, because NCM endosomes are primarily fated for degradation [49], we reasoned that EGFR would experience increased degradation at longer EGF exposure times in 1,3,4-*O*-Bu_3_ManNAc-treated cells compared to untreated controls. Therefore the time-dependent increase in the degradation of EGFR we observed (e.g., at 30 min (Figure 7A) or 60 min (Figure 7B)) further support the hypothesis that 1,3,4-*O*-Bu_3_ManNAc increases internalization *via* a shift to NCM endocytosis.

### ERK1/2- and AKT-driven signaling do not respond to 1,3,4-*O*-Bu_3_ManNAc treatment

**Figure 8:**
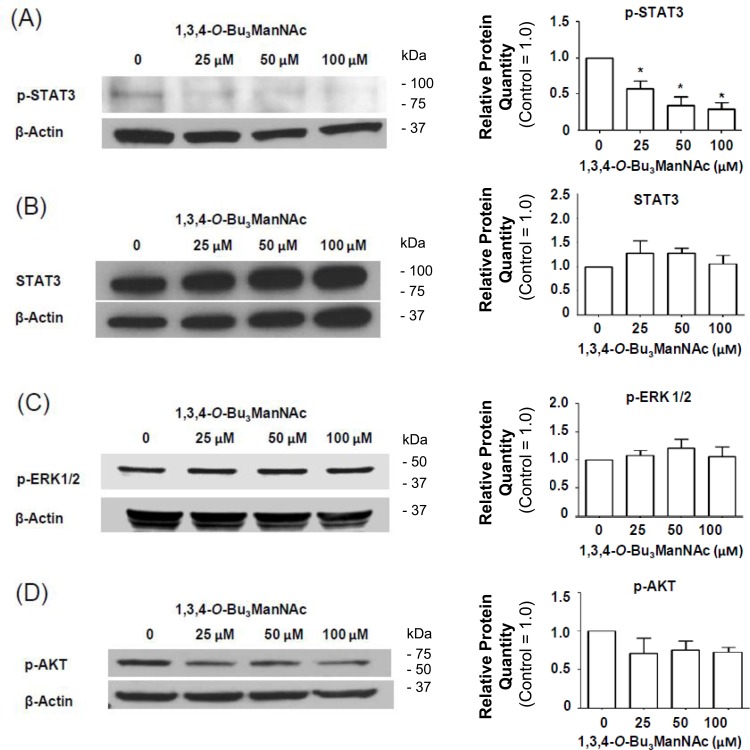
Impact of 1,3,4-*O*-Bu_3_ManNAc on downstream signaling of STAT and ERK1/2 Western blots of lysates from SW1990 cells treated with 1,3,4-*O*-Bu_3_ManNAc. The treated (and control) cells were subsequently exposed to 10 ng/mL of EGF for 2.0 min and (**A**) the amount of phosphorylated STAT3 significantly decreased (**B**) without a corresponding decrease in STAT3 levels (**B**). At the same time, phosphorylation of ERK1/2 (**C**) and AKT (**D**) were not affected. Each experiment includes at least three biological replicates and the blots were quantified using Image J with data expressed as mean ± standard error mean (SEM). * indicates a p value of < 0.05.

We next sought to gain insight into the impact of NCM-shifted internalization on the signaling activity of EGFR in 1,3,4-*O*-Bu_3_ManNAc treated SW1990 cells. This receptor must be phosphorylated to initiate signaling and we previously showed that changes in p-EGFR levels in 1,3,4-*O*-Bu_3_ManNAc treated SW1990 cells, while modest, were amplified downstream in stronger inhibition of STAT3 phosphorylation (p-STAT3) [1]. Notably, the previously-reported decrease in p-STAT3 levels (which were reproduced in the current experiments, Figure 8A) was confirmed to not be due to a change in overall STAT3 levels (Figure 8B), providing additional evidence that decreased p-STAT3 legitimately reflected attenuation of EGFR signaling pathway activation upon 1,3,4-*O*-Bu_3_ManNAc treatment and not changes in the overall levels of this protein.

In addition to STAT3, p-ERK1/2 and p-AKT can also be activated by EGFR; accordingly, we investigated whether these additional downstream effectors of p-EGFR signaling were reduced in 1,3,4-*O*-Bu_3_ManNAc-treated cells. In these experiments, EGFR-driven signaling *via* the ERK1/2 and AKT pathways was monitored by measuring phosphorylated ERK1/2 (p-ERK1/2) and phosphorylated AKT (p-AKT) using western blot analysis. These experiments showed that no statistically significant change occurred for p-ERK1/2 (Figure 8C) or p-AKT (Figure 8D). The minimal response of p-ERK1/2 and p-AKT in 1,3,4-*O*-Bu_3_ManNAc-treated cells can be explained by activation of ERK1/2 and AKT *via* RAS [35, 55]; mutations that constitutively activate RAS signaling have long been associated with non-small cell lung cancer and metastatic colorectal cancer [56] and now have been linked to pancreatic cancer. Consistent with this information, the RAS pathway is constitutively activated in the SW1990 cell line used in this study [57], which represents the clinical situation for a large majority (e.g., ≥ 81% [58, 59]) of pancreatic cancer patients. Activation by these alternate pathways negates the impact of reduced p-EGFR levels on ERK1/2 and AKT in 1,3,4-*O*-Bu_3_ManNAc-treated SW1990 cells. By contrast, because STAT3 activity is not primarily driven by RAS signaling [60, 61] (although linked to RAS in a parallel and complementary manner [62]), we reasoned that inhibition of STAT3 by 1,3,4-*O*-Bu_3_ManNAc nevertheless could provide therapeutic benefit even in cells with constitutively active RAS. To test this premise, we next evaluated the expression of selected p-STAT3-driven oncogenes in 1,3,4-*O*-Bu_3_ManNAc-treated cells.

### Downstream STAT3-driven genes respond to analog-mediated p-EGFR inhibition

**Figure 9:**
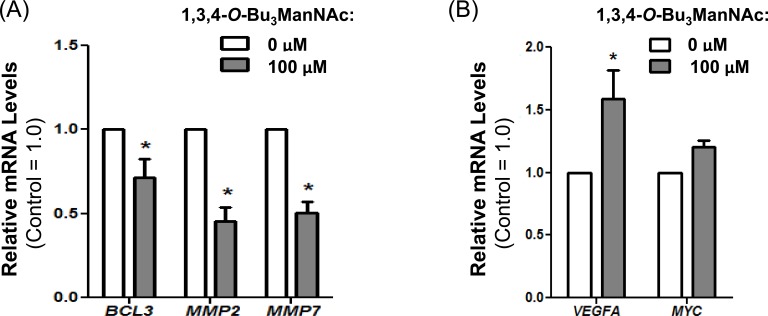
RT-PCR analysis of SW1990 cells treated with and without 100 μM of 1,3,4-*O*-Bu_3_ManNAc showed (**A**) a significant decrease in expression of STAT3 associated genes BCL3, MMP2 and MMP7 whereas (**B**) VEGFA and MYC, which can also be regulated by STAT3, showed increased expression (VEGFA) or was not affected (MYC). At least 3 biological replicates were carried out for each experiment with data expressed as mean ± standard error mean (SEM). * indicates a p value of < 0.05.

Although not all downstream effectors driven by p-EGFR are inhibited by 1,3,4-*O*-Bu_3_ManNAc treatment in SW1990 cells (e.g., ERK1/2 and AKT signaling do not respond as described above), we found that several important oncogenes activated by p-STAT3 were successfully inhibited by treatment with this sugar analog. In particular, the reduction in p-STAT3 (Figure 8A) was correlated with decreased expression of *BCL3, MMP2*, and *MMP7* (Figure 9A). This downstream modulation of several p-STAT3-driven genes that contribute to cancer progression demonstrates that even modest changes in the activity of surface receptors due to altered glycosylation have potential therapeutic benefit.

Conversely, offsetting factors – including the negligible response of ERK1/2 and AKT and other EGFR-responsive genes including *MYC* and *VEGFA* [63] (Figure 9B) to attenuated p-EGFR levels in SW1990 cells – suggest that a compound such as 1,3,4-*O*-Bu_3_ManNAc is unlikely to comprise a “stand alone” drug for advanced stage pancreatic cancers. Indeed, several “glycosylation-only” EGFR-targeting therapies have recently been judged to be ineffective as cancer therapies (34). Instead, as we recently reported [1], synergy between otherwise ineffective TKIs (e.g,. gefitinib and erlotinib) and 1,3,4-*O*-Bu_3_ManNAc holds promise for combination therapy in drug resistant cancers. The mechanism behind the observed synergy was unclear, however, especially considering that there are very few strategies where drug synergy is achieved by directing multiple drugs against the same biomolecular target (in this case, EGFR). To address this issue, we investigated how two marginally effective EGFR-targeting strategies achieve synergy when used in combination in SW1990 cells [1].

### Synergy between 1,3,4-*O*-Bu_3_ManNAc and erlotinib

**Figure 10:**
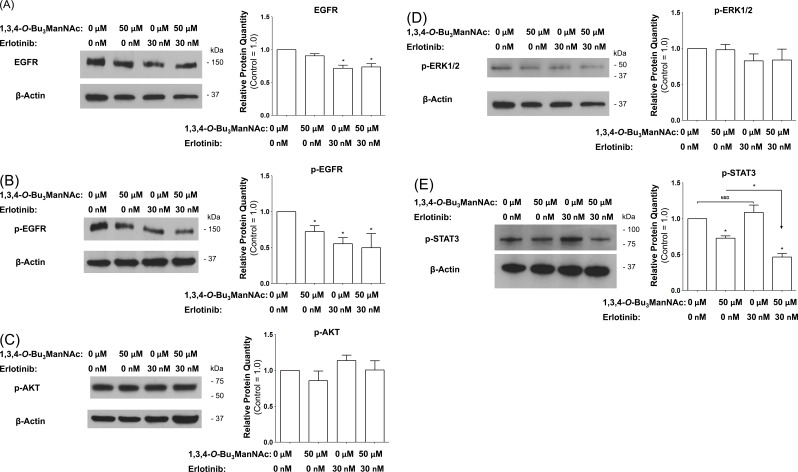
Western blot analysis of SW1990 cells treated with 50 μM 1,3,4-*O*-Bu_3_ManNAc, 30 nM of erlotinib, or both compounds in combination showed (**A**) EGFR levels that are not affected by the analog alone are decreased by erlotinib. (**B**) EGFR phosphorylation is inhibited by both compounds individually as well as in combination. (**C**) and (**D**) p-AKT and p-ERK1/2 levels, respectively, are not significantly affected by any of the treatment conditions. (**E**) p-STAT3 levels are inhibited by the analog but are not affected by erlotinib; however the combination of the two compounds leads to significantly lower p-STAT3 levels when compared to the analog alone. At least 3 biological replicates were carried out for each experiment with data expressed as mean ± standard error mean (SEM). * indicates a p value of < 0.05.

To gain insight into synergy between 1,3,4-*O*-Bu_3_ManNAc and erlotinib [1], various downstream components of EGFR signaling were analyzed via western blots in SW1990 cells treated with 1,3,4-*O*-Bu_3_ManNAc, erlotinib, or both. First, as expected from previous experiments, 1,3,4-*O*-Bu_3_ManNAc alone did not significantly affect EGFR levels (Figure 10A). In this experiment, erlotinib treatment decreased EGFR levels but cotreatment with 1,3,4-*O*-Bu_3_ManNAc did not enhance this effect, indicating a lack of synergy. Next, EGFR phosphorylation was tested and 1,3,4-*O*-Bu_3_ManNAc (as expected from previous results) as well as erlotinib decreased p-EGFR but again cotreatment did not have a synergistic effect (Figure 10B). Moreover, none of the treatment conditions significantly altered either p-AKT or p-ERK1/2 levels (Figure 10C and Figure 10D respectively), again leaving synergy between 1,3,4-*O*-Bu_3_ManNAc and erlotinib unexplained.

Instead, synergy between 1,3,4-*O*-Bu_3_ManNAc and erlotinib depended on modulation of STAT3. Specifically, levels of p-STAT3 – although not affected by erlotinib when used by itself – experienced an amplified decrease upon exposure to 1,3,4-*O*-Bu_3_ManNAc compared to treatment with the sugar analog by itself (Figure 10E). Of the many conditions tested, the ability of co-treatment to amplify p-STAT3 inhibition provides an explanation for the synergy observed between 1,3,4-*O*-Bu_3_ManNAc and TKI drugs; we emphasize that other factors such as control of STAT3 by cytokine, GPC, or toll-like receptors, which is beyond the scope of the current study, may also contribute to the synergy.

## DISCUSSION

This report builds on previous studies where we characterized glycosylation in advanced stage pancreatic cancer SW1990 cells treated with 1,3,4-*O*-Bu_3_ManNAc to understand how metabolic flux-driven increases in sialic acid contribute to cancer progression [4, 5]. Our focus on pancreatic cancer was motivated by poor prognoses for this disease, which has a five year survival rate of only ∼4% [64]; we reasoned that gaining a better understanding of glycosylation could be valuable for devising new treatment strategies. This premise was supported by our discovery that 1,3,4-*O*-Bu_3_ManNAc sensitized SW1990 cells to the EGFR-targeting TKI drugs erlotinib and gefitinib [1].

We first sought a mechanistic understanding of how 1,3,4-*O*-Bu_3_ManNAc and the resultant increase in sialylation (Figure 1 and [4]), dampened EGFR signaling. Based on recent reports by others that sialyltransferase over-expression diminishes EGFR signaling by inhibiting dimerization of this glycoprotein in several cell lines [29, 33, 34], we first tested whether increased sialylation achieved through 1,3,4-*O*-Bu_3_ManNAc treatment of SW1990 cells had a similar effect. Experiments following the protocols described by Yen and coauthors [34] showed a slight trend towards reduced dimerization but the results were not statistically significant; together with data shown in Figure 2 where there was no difference in the on/off rate of EGF binding in treated or untreated cells, we concluded that the previously-reported dimerization mechanism offered, at most, only a partial explanation for the impact of 1,3,4-*O*-Bu_3_ManNAc on EGFR activity in SW1990 cells. This disparity – along with indications that cell surface trafficking of EGFR was involved in cellular responses to this sugar analog (Figure 3) – led us to explore additional mechanisms by which sialylation modulates EGFR signaling. One such plausible mechanism could be the increased expression of polysialic acid, which has been associated with both increased metabolic flux through the sialic acid pathway [65] and metastatic pancreatic cancer [66]. However, in the case of SW1990 cells which metastasize to the kidney (as compared to metastasis to neural tissue where PSA is required [66]), there is no evidence that PSA is involved in SW1990 cell metastasis, consistent with our extensive previously-reported evaluation of N-linked glycans in this cell line [5]. Based on this evidence that PSA is not involved in the changed biological activity of SW1990 cells upon treatment with 1,3,4-*O*-Bu_3_ManNAc, we sought (and verified) alternative mechanisms as described next.

Based on our previous demonstration that 1,3,4-*O*-Bu_3_ManNAc approximately doubles overall levels of α2,6-sialic acid in SW1990 cells [4, 5] and the current evidence that α2,6-sialylation of EGFR also increases, albeit moderately (Figure 1), the galectin lattice provides an attractive complementary mechanism to the dimerization hypothesis. The basis for evoking the galectin lattice – as outlined in Figure 4 – to explain the impact of 1,3,4-*O*-Bu_3_ManNAc in SW1990 cells is that α2,6-sialic acid inhibits galectin affinity for underlying galactose/GalNAc epitopes [48]; the resulting negative regulation of the lattice reduces surface display of EGFR by promoting internalization, which reduces signaling potency [45]. Building on data from earlier experiments, (e.g., our prior publications [1, 4]), Figures 1 and 2 of this paper, and support from modeling simulations (see the Supplemental Material), we showed (e.g., in Figure 5) that increased internalization consistent with attenuation of the galectin lattice occurs.

Mechanistically, the galectin lattice directly interacts with cell surface glycoproteins and affects their trafficking, activity, and signaling potency [67]. This mechanism provides a plausible explanation for the changes to EGFR activity and trafficking observed in 1,3,4-*O*-Bu_3_ManNAc-treated cells because, by increasing global cell surface sialylation by ∼2-fold [4], this analog is expected to reduce galectin binding to cell surface glycans thereby increasing the surface mobility of cell surface receptors such as EGFR. This idea is consistent with evidence that micron-scale membrane domain organization structure influences EGFR mobility on the cell surface through a glycosylation-based mechanism [45] and the growing understanding of how the galectin lattice represents an important layer of membrane organization. Indeed, the lattice has been described as a “gel-like polymer that regulates glycoprotein distribution” that controls diffusion, complex formation and domain interactions in the plasma membrane [68].

Another significant aspect of the current study is that we outline downstream consequences of attenuation of the galectin lattice with 1,3,4-*O*-Bu_3_ManNAc, which not only increases the rate of EGFR internalization but shifts it to a new mode of endocytosis. Specifically, internalization of this receptor shifted from a clathrin-mediated mechanism towards NCM endocytosis (Figure 6) upon increased sialylation and this shift led to enhanced degradation of EGFR (Figure 7). Together, these results provide an alternative and complementary mechanism to the dimerization hypothesis associated with sialyltransferase-mediated increases in sialylation and helps explain how increased flux-based sialylation attenuates EGFR signaling and holds anti-cancer potential for treating drug resistant pancreatic cancer despite resistance to TKIs or constitutive Ras activation (Figure 8 and references [69, 70]). To elaborate briefly, a shift towards NCM endocytosis rapidly ablates EGFR activity compared to “normal” clathrin-coated internalization where signaling is maintained or even enhanced upon endocytosis; for example clathrin-mediated endocytosis is essential for MAPK activation [71]. Similarly, EGFR activity can continue from within endosomes [72, 73] and signaling emanating from clathrin-coated endosomal vesicles can be adequate to promote cell survival even in the absence of surface signaling cues [74]. Therefore the ability of 1,3,4-*O*-Bu_3_ManNAc to redirect EGFR trafficking away from this activating mode of internalization provides important insights into how this compound attenuates signaling of this oncogene.

Altered vesicular trafficking has particularly important ramifications for STAT3-driven gene expression because clathrin-mediated endocytosis supports cytoplasmic transport of STAT3 to the nucleus [75]. This observation helps explain the strong diminution of expression of STAT3-associated genes *BCL3*, *MMP2* and *MMP7* in 1,3,4-*O*-Bu_3_ManNAc treated cells (Figure 9A) despite maintenance of other aspects of EGFR signaling. Furthermore, synergy between 1,3,4-*O*-Bu_3_ManNAc and erlotinib was linked to pSTAT3 (Figure 10E) insofar as cells treated with erlotinib alone sustain STAT3 activity despite lower overall levels of EGFR and p-EGFR. Instead, for the reduced levels of EGFR caused by erlotinib to be manifest in downstream activity, co-treatment with 1,3,4-*O*-Bu_3_ManNAc was needed to shift clathrin-mediated internalization towards NCM endocytosis and rapid inactivation rather than prolonged signaling. From a practical perspective, this synergy holds intriguing potential to combat drug-resistant pancreatic cancer, which remains virtually untreatable to date.

In conclusion, this report provides evidence that flux-driven sialylation reduces EGFR signaling by masking galectin binding epitopes [76]; this strategy offers an alternative, and potentially more facile, method to attenuate lattice effects compared to intervening in the production of highly-branched N-glycan structures that function as galectin binding epitopes [77–79]. Our results further show that attenuation of the galectin lattice directs EGFR trafficking away from clathrin-mediated internalization (which relies on galectin binding [80]) towards NCM endocytosis, which explains the inhibitory effects of 1,3,4-*O*-Bu_3_ManNAc on downstream pSTAT-activated genes and synergy with TKIs. However, due to the complexities of both glycosylation and cell signaling, we mention several caveats. First, while we did not obtain evidence to support the previously-reported dimerization hypothesis to explain the effect of altered sialylation on EGFR in SW1990 cells, our experiments did not rule out this mechanism for other cell types or for genetic manipulation of sialylation. Second, yet another mechanism by which increased sialylation could in theory suppress EGFR signaling is by increasing cellular levels of ganglioside GM3 [81]. Of course altering the galectin lattice, and by extension the bulk fluidic properties of the plasma membrane, almost certainly affects additional pathways beyond EGFR signaling [78, 79, 82]. Therefore, although we present evidence that 1,3,4-*O*-Bu_3_ManNAc acts via EGFR through galectin lattice effects, cell-level behavior (e.g., synergy with TKI drugs) most likely has additional inputs beyond the scope of this study. Nevertheless we emphasize that this report describes the important and novel finding that a pharmacologically relevant small molecular (1,3,4-*O*-Bu_3_ManNAc) provides the same functional benefits of less translatable genetic approaches in sensitizing drug-resistant cancer cells to TKI inhibitors and provides mechanistic insight into this phenomenon. We believe that this new information provides an important scientific foundation for both continued basic science investigations into the underlying mechanisms but also provides impetus for clinical translational of this strategy that holds promise for prolonging the effectiveness of existing cancer drugs (e.g., erlotinib and gefitinib).

## MATERIALS AND METHODS

### Cell culture and incubation with 1,3,4-*O*-Bu_3_ManNAc

SW1990 (ATCC^®^ CRL-2172) cells were grown in Dulbecco's Modified Eagle Medium (DMEM) supplemented 10% with heat-inactivated fetal bovine serum (FBS) and 1.0% of a 100x pen/strep antibiotic solution (Invitrogen). Cells were maintained at 37°C in a humidified, 5% CO_2_-containing atmosphere. 1,3,4-*O*-Bu_3_ManNAc was synthesized and characterized as previously described [2, 3] and stored lyophilized at -80°C. Stock solutions (100 mM) were made in ethanol (EtOH). For analog treatment, cells typically were plated in 6-well tissue culture plates in 2.0 mL of culture media at a density of 300,000 cells/well and the appropriate volume of 1,3,4-*O*-Bu_3_ManNAc was added to each well to achieve the desired analog concentrations; the identical volume of ETOH (always less than 10 μL/mL) was added to each well in each experiment to ensure that all cells were exposed to the same amount of solvent as those treated with the highest concentration of analog. Cells were typically incubated for 48 h with the sugar analogs; in certain experiments (as indicated below) the first 24 h of incubation was carried out in complete media and the cells were serum starved for the final 24 h before analysis following published protocols for monitoring EGFR phosphorylation and activation [29].

### Western blot analysis

Proteins obtained from SW1990 cells were analyzed by western blots after the cells were incubated with 1,3,4-*O*-Bu_3_ManNAc, erlotinib or both for 48 h including, as described above, serum starvation for the last 24 h and exposure to 10 ng/mL recombinant human EGF (Peprotech AF-100-15) in PBS for 2.0 min, 30 min or 60 min. Proteins were collected and quantified using the BCA assay (ThermoFisher) after which time normalized aliquots were separated using polyacrylamide gel electrophoresis and then were immunodetected using the following commercial antibodies: anti-phospho-EGFR (p-EGFR, Tyr1068 Cell Signaling #4267), anti-EGFR (D33B1, Cell Signaling, #4267), anti-STAT3 (STAT3, 79D7, Cell Signaling #4904), anti-phospho-STAT3 (p-STAT3, Tyr705, Cell Signaling #9131), anti-phospho-AKT (p-Akt, Ser473 (D9E) Cell Signaling #4060), anti-phospho-ERK1/2 (p-ERK1/2 Try202/204, Cell Signaling, #9101), anti-β-actin (Sigma-Aldrich) and HRP-linked anti-rabbit antibody (Cell Signaling). Protein bands were quantified using the ImageJ software. Where necessary, blots were stripped with Gentle Review Stripping Buffer (Amresco), reblocked and analyzed. In cases where blots were re-analyzed, the control samples for the data presented (e.g., for p-EGFR and p-AKT in Figure 7B) are in some cases the same because, out of the multiple samples available for presentation, the ones that were the most visually representative of the “Image J” quantification were shown.

### EGFR immunopurification and characterization of sialylation

Cells were incubated for 48 h with 100 μM 1,3,4-*O*-Bu_3_ManNAc, rinsed in ice cold PBS, collected using cell scrapers, and resuspended in 0.5 mL of ice cold cell lysis buffer (Cell Signaling). Samples were sonicated on ice three times for 5 s each and then samples were centrifuged for 10 min at 4°Cat 14,000*g.* Protein from control and treated cells were collected from the supernatant, quantified using the Pierce 660 nm protein assay (Thermo Scientific); protein levels were then normalized to 1.0 mg/mL. EGFR from control and treated samples was then immunopurified using Sepharose bead conjugated EGFR mAb (Cell Signaling) following the manufacturer's protocol. After purification, the samples were divided in two with half of the samples boiled in loading buffer for 10 min and then analyzed for total EGFR protein levels by western blotting as described above. HRP-linked SNA-1 Lectin (EY Laboratories) was also used to stain western blots of immunopurified EGFR to determine the levels of α2-6 linked sialic acid. Band intensities were quantified using ImageJ software and normalized to EGFR levels.

### Fluorescent assisted carbohydrate electrophoresis (FACE)

The other half of the immunopurified EGFR samples were digested with sialidase (P0722L, New England BioLabs), wherein 10 μL of immunopurified EGFR on sepaharose beads was incubated with 200 units of sialidase in a 100 μL reaction volume for 48 h at 37°C. After sialidase digestion, the samples were centrifuged at 14,000*g* and the amount of sialic acid released into the supernatants was determined by FACE following an established protocol (83,84). Briefly, 50 mg graphitized carbon columns were prepared and activated with 80% acetonitrile, 0.1% v/v trifluoroacetic acid (TFA) using three 1.0 mL washes and were then equilibrated with five 1.0 mL milli Q water washes under vacuum. The supernatants were then loaded onto the columns and the columns were washed five times with 1.0 mL of milliQ water under vacuum after which the released sialic acids were eluted under gravity using 1.0 mL of 25% acetonitrile, 0.1% v/v TFA. The samples were then lyophilized, resuspended in 150 μL of milli Q water, transferred into fresh 1.5 mL eppendorf tubes, and lyophilized again. These samples, along with sialic acid standards, were then labeled with 40 μL of a 6.25 mM 2-aminoacridone (Carbosynth) solution in DMSO overnight at 37°C.

A gel solution was prepared with 500 mL of 40% acrylamide (BioRad), 100 mL tris-acetate (400 mM, pH 7.0), 370 mL milliQ water and 25 mL of glycerol. An aliquot of the gel solution (5 mL) was then mixed with 25 μL of 10% ammonium persulfate and 5 μL of TEMED (BioRad) and poured into preassembled casting plates with a 0.75 mm well comb. After 7.5 min the combs were removed and the gels were transferred into the gel apparatus (BioRad) and the apparatus was filled with 1X tris-borate EDTA (BioRad). The gel apparatus was then placed on ice for 2.0 h. An aliquot of each sample (2.0 μL) was then loaded onto the gel and the gel was run at 500 V for 40 min on ice. The gel was then transferred onto a visi-blue benchtop variable UV transilluminator and imaged. Band intensities were then quantified using ImageJ software and normalized to the EGFR levels measured in the western blots described above.

### Confocal microscopy for cell surface EGFR measurement

Cells were incubated for 48 h with 1,3,4-*O*-Bu_3_ManNAc with serum starvation over the last 24 h. Cells were washed in 1.0 mL of PBS and then fixed in 3.7% formaldehyde for 10 min. Cells were blocked with 5% bovine serum albumin (BSA) in PBS for 1.0 h and then incubated overnight at 4°C with Alexa Fluor 488-conjugated EGFR mAb (Cell Signaling). Nuclei were stained with DAPI. After three washes in PBS, the cells were imaged on Zeiss AxioObserver with 780-Quasar confocal module & FCS. Gross fluorescence was determined for Alexa Fluor 488-conjugated EGFR mAb and DAPI for each image using ImageJ software. The relative fluorescence of each 1,3,4-*O*-Bu_3_ManNAc-treated sample was determined by normalizing Alexa Fluor 488-conjugated EGFR mAb fluorescence to DAPI fluorescence and then normalizing to control samples not treated with analog.

### Fluorescent recovery after photobleaching (FRAP) assays

Cells were incubated for 48 h with 1,3,4-*O*-Bu_3_ManNAc with serum starvation over the last 24 h. The cells were washed in Live Cell Imaging Solution (Life Technologies) supplemented with 1.0% bovine serum albumin (BSA) and 20 mM glucose and then incubated at 37°C with 2.0 μg/mL of Alexa Fluor 488-conjugated EGF (Life Technologies). Cells were then analyzed by fluorescence recovery after photobleaching technique (FRAP) [85, 86] under two conditions. In the first approach, which was adapted from Sprague et al. who describe the use of FRAP for analysis of binding interactions [87]; unbound Alexa Fluor 488-conjugated EGF was maintained in the bath, which allowed us to measure EGF on/off rate (as shown in Figure 2C). In the second set of experiments, unbound EGF was removed by washing before imaging, in which case membrane fluidity was measured (Figure 5A). Cells were incubated at 37°C for the duration of the FRAP experiments. Then, using a Zeiss 780 FCS Confocal Microscope together with a 488 nm Argon ion laser for excitation of Alexa Fluor 488 we monitored emissions at 525 nm. The laser intensity was adjusted to obtain a 75 % loss in fluorescence in a rectangular 3.0 by 1.0 μm photobleached region on the apical focal plane of the cell membrane; for rapid bleaching high laser intensities were used for a single bleaching scan (0.278s). Multiple regions were imaged pre- and post- photobleaching using low laser intensities and recovery fluorescence in the selected regions was tracked over time.

The fluorescent intensity measured at each time point (I(t)) was then converted to a normalized fluorescent intensity (NFI(t)) normalized using the following equation: Normalized Fluorescent Intensity= [{I(t)-I(post bleach)}/I(prebleach)]/I(post recovery) The NFI was then plotted against time and fit to a one phase exponential association curve using the GraphPad Prism 6 software (GraphPad Software, Inc.; La Jolla, CA). From the fit of the curves, time constants for half recovery were derived (t_0.5_).

### Mathematical modeling of EGFR trafficking

Based on evidence that 1,3,4-*O*-Bu_3_ManNAc-mediated changes to sialylation in SW1990 cells impact EGFR by a different mechanism than previously observed for sialyltransferases, we proposed that this compound affected EGFR trafficking *via* changes to the galectin lattice [45]. Before undertaking additional experiments to support this hypothesis, we implemented a MATLAB model of the surface dynamics and recycling kinetics of this receptor [44] to gain support for the galectin lattice mechanism (or to rule it out) and to evaluate whether any competing, and perhaps more compelling, hypotheses existed. As described in detail in the Supplemental Material, the computational model was consistent with the galectin lattice hypothesis; it did not support any alternative mechanisms; and it helped guide investigation of different modes of internalization as presented below.

### Internalization assays

Cells were incubated for 48 h with 1,3,4-*O*-Bu_3_ManNAc with serum starvation over the last 24 h. The cells then were washed with PBS, treated with enzyme free cell dissociation buffer (Life Technologies) until they detached from the culture plate, collected, and counted and cell numbers were normalized using the Beckman Z2 cell coulter counter. Cells were then washed twice in Live Cell Imaging Solution (Life Technologies) supplemented with 1.0% bovine serum albumin (BSA) and 20 mM glucose and treated with 0.5 μg/mL of filipin (Sigma Aldrich) or 100 mM of lactose (Carbosynth) for 60 min. Cells were then incubated at 37°C for 30 min with 2.0 μg/mL of Alexa Fluor 488-conjugated EGF (Life Technologies). Cells were washed three times, followed by acid washing for 5.0 min with 0.2 M glycine (pH 2.5), washed thrice and finally analyzed using flowcytometry with an Accuri C6 Flowcytometer. The cell population of interest was gated appropriately and 10^4^ cells falling within the gated area were measured and used to determine the mean fluorescence of the cell population; the histograms for these experiments are shown in Figure S6 in the Supplemental Material.

### Lectin binding assays

Cells were incubated for 48 h with 1,3,4-*O*-Bu_3_ManNAc. The cells were washed with PBS, treated with enzyme free cell dissociation buffer (Life Technologies) until they detached from the culture plate, collected, and counted and cell numbers were normalized using the Beckman Z2 cell coulter counter. Cells were then washed twice in PBS. Cells were then incubated at room temperature for 120 min with 5.0 μg/mL of Flourescein-labeled RCA lectin (Vector Laboratories). Cells were washed three times in PBS and analyzed using flowcytometry with an Accuri C6 Flow cytometer. The cell population of interest was gated appropriately and 10^4^ cells were used to determine mean fluorescence.

### Confocal microscopy for endosome sizing

Cells were incubated for 48 h with 1,3,4-*O*-Bu_3_ManNAc with serum starvation over the last 24 h. Cells were then washed in Live Cell Imaging Solution (Life Technologies) supplemented with 1.0% bovine serum albumin (BSA) and 20 mM glucose. Cells were then incubated at 37°C for 10 min or 30 min with 2.0 μg/mL of Alexa Fluor 488-conjugated EGF (Life Technologies). Cells were then fixed, nuclei were stained with DAPI and then imaged on Zeiss AxioObserver with 780-Quasar confocal module & FCS. Endosome size was estimated using ImageJ software based on published procedures (88,89).

### Quantitative reverse transcription-polymerase chain reaction (qRT-PCR)

Total RNA was isolated using Trisol^®^ reagents (Gibco BRL) and reversed transcribed using the high capacity RNA-to-cDNA kit (Applied Biosystems). PCR amplifications were performed using the following TaqMan® Gene Expression Assays from Applied Biosystems: *BCL3* (Assay ID: Hs00180403_m1), MYC (Assay ID: Hs00153408_m1), *VEGFA* (Assay ID: Hs00900055_m1), *MMP2* (Assay ID: Hs01548727_m1), *MMP7* (Assay ID: Hs01042796_m1) and *GAPDH* (Assay ID: Hs03929097_g1). **q**RT-PCR was performed using the Step-One Plus Real-Time PCR system (Applied Biosystems) with the thermocycling conditions of 50°C for 2.0 min, 95°C for 10 min followed by 40 cycles of 95°C for 15 s and 60°C for 1.0 min.

### Statistical analysis

Data was expressed as means ± standard error (SEM). Statistical significance was determined using one way ANOVA with a Dunnett's post-test to compare means of different samples with the control or a Bonferroni post test to compare specific pairs of columns. The null hypothesis was rejected in cases where p-values were < 0.05.

## SUPPLEMENTARY MATERIAL FIGURES




