# Supplementary material for: Metabolic flux-driven sialylation alters internalization, recycling, and drug sensitivity of the epidermal growth factor receptor (EGFR) in SW1990 pancreatic cancer cells
Source: Oncotarget. 2016 Aug 24;7(41):66491–511. doi: 10.18632/oncotarget.11582 (PMC5341816; doi:10.18632/oncotarget.11582)
Supplement: Supplementary file 1 [file oncotarget-07-66491-s001.pdf]

Metabolic flux-driven sialylation alters internalization, recycling, and drug sensitivity of the epidermal growth factor receptor (EGFR) in SW1990 pancreatic cancer cells

Supplementary Material

This document contains the following supporting information for the main manuscript:

1) Clarification of the sialylation status of EGFR sialylation in SW1990 cells
2) Computational modeling of EGFR trafficking
3) Flow cytometry histograms to support **Figure 5** of the main manuscript

1. SIALYLATION OF SW1990 CELLS AND EGFR:

In our previous work we extensively characterized the impact of the "high flux" ManNAc analog 1,3,4-*O*-Bu_3ManNAc on sialylation of SW1990 cells; this data provides a basis for the current study. For the interested reader, background information on this compound is provided in several of our publications that describe how this compound increases sialylation by a metabolic flux-based mechanism [1-5] with only minor changes to gene expression compared to other similar compounds, e.g., 3,4,6-*O*-Bu_3ManNAc [1,6]. Here, we reproduce results showing overall changes to sialic acid in SW1990-treated cells (in **Figure S1**) and also provide the unpublished "medium confidence" (i.e., basically, > 95% but < 99%) results from the glycoproteomics experiments (in the accompanying excel file titled **Supp Data_Glycoproteomic glycosite evaluation of SW1990 cells.xls**, with the relevant data for EGFR provided in line 216, and the increase in sialylation of 2.11 given in column AR (for more detail, please refer to the parent publication for this data, Almaraz *et al.*,[4]).

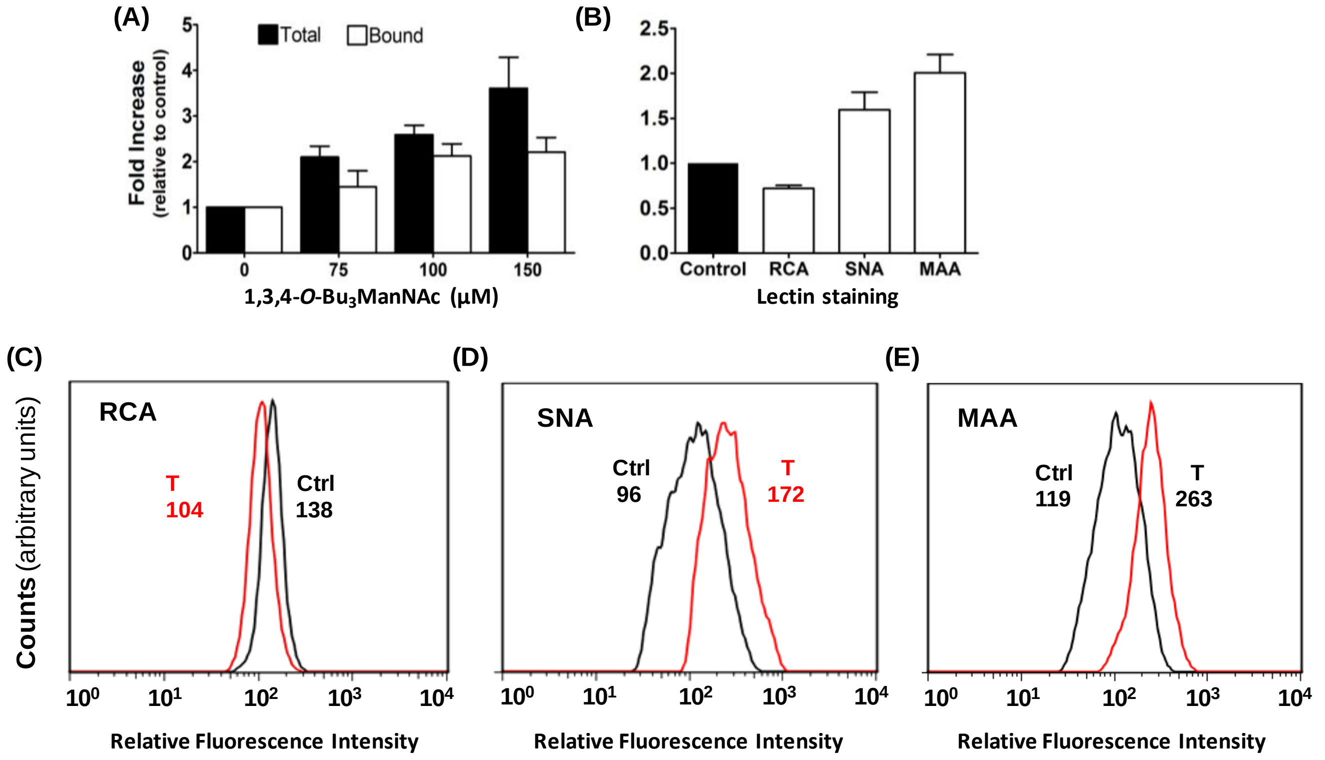

<u>**Supplemental Figure S1**</u>: **Changes in intracellular and surface sialic acid in 1,3,4-*O*-Bu_3ManNAc-treated SW1990 cells** (adapted from Almaraz *et al*., [4]). (**A**) Total (which includes all forms of sialic acid found within a cell, black bars) and "glycoconjugate-bound" (which also includes soluble CMP-sialic acid, white bars) sialic acid was measured by using the periodate resorcinol assay in cells incubated with the indicated concentrations of 1,3,4-*O*-Bu_3ManNAc for 2 days. Cells treated with 100 µM 1,3,4-*O*-Bu_3ManNAc for 2 days were analyzed by lectin binding with results summarized in (**B**) and representative flow cytometry data shown in (**C**) for ricin agglutinin (RCA), (**D**) *Sambucus nigra* agglutinin (SNA), and (**E**) *Macckia amurensis* agglutinin (MAA) flow cytometry. The error bars represent the S.D. of three different experiments.

This data supports the galectin lattice mechanism described in the main manuscript insofar as SNA binding represents levels of α2,6-linked sialic acids, which inhibit access to the underlying galactose/GalNAc residues bound by galectins and – as shown above – RCA. Of note, these global changes to sialylation are larger in magnitude than those observed for immunopurified EGFR, which was newly analyzed in the current study (with data provided in **Figure 1** of the main manuscript).

2. Mathematical modeling of EGFR trafficking:

Introductory Comments and Rationale for Model Implementation

The effect of parameter changes in the western blot and saturation binding experiments presented in our previous work (7) and surface localization assays described in the main manuscript (**Figure 2**) on EGFR trafficking were analyzed by a macroscopic level model (8) that contained five components: ligand binding, synthesis, internalization, degradation, and recycling **(Figure S2**).

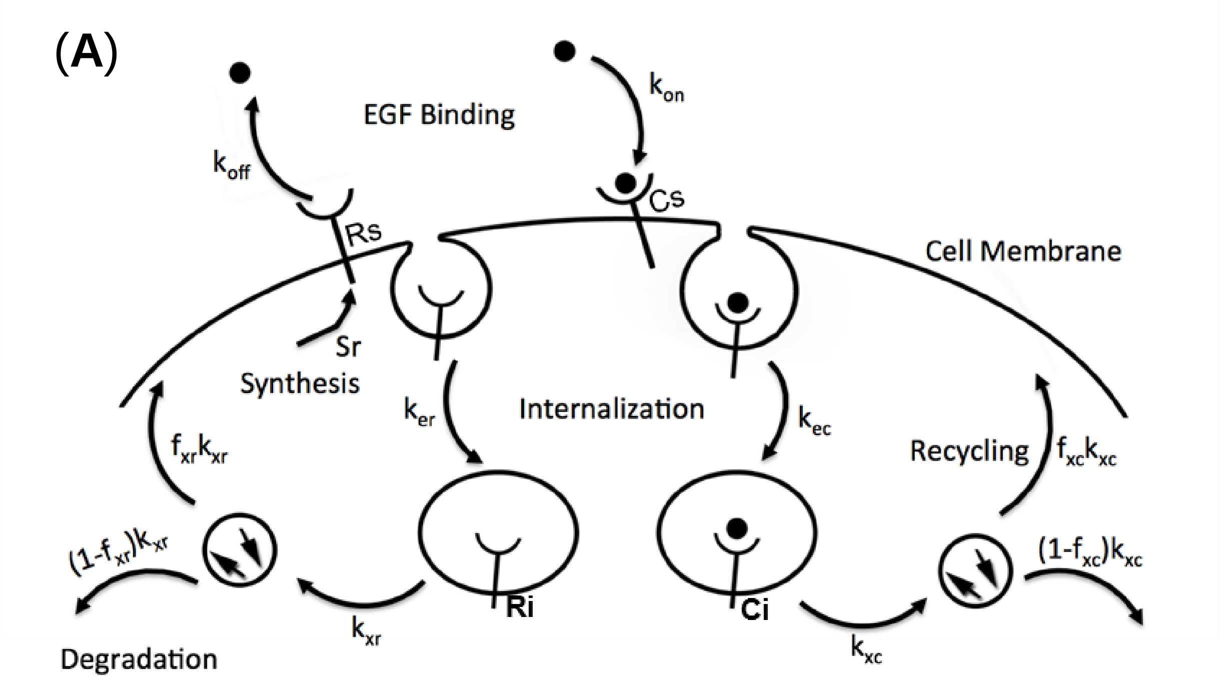

(B)

$$\frac{\mathbf{dR_s}}{\mathbf{dt}} = -k_{on}LR_s + k_{off}C_s - k_{er}R_s + k_{xr}(f_{xr})R_i + S_r$$

$$\frac{\mathbf{dC_s}}{\mathbf{dt}} = +k_{on}LR_s \_ k_{off}C_s - k_{ec}C_s + k_{xc}(f_{xc})C_i$$

$$\frac{\mathbf{dR_i}}{\mathbf{dt}} = +k_{er}R_s - k_{xr}(f_{xr})R_i - k_{xr}\ (1 - f_{xr})R_i$$

$$\frac{\mathbf{dC_i}}{\mathbf{dt}} = +k_{ec}C_s - k_{xc}(f_{xc})C_i - k_{xc}(1 - f_{xc})C_i$$

__**Supplemental Figure S2**__. **Mathematical model of EGFR trafficking**. **(A)** The general schematic (adapted from [8], with additional details provided below for modeling EGFR trafficking is shown, with the equations used for model output predictions given in (**B**).

The baseline parameters used in this study were adapted from a model of human mammary epithelial cells described by Hendricks *et al*., [8]. We note that these parameters were derived from a different cell line and therefore were not expected to be quantitatively predictive in the currently-used SW1990 line; consequently the model was not expected to (necessarily) be able to predict subtle differences between closely matched drug candidates, for example. However because EGFR trafficking across human cell types utilizes the five basic components of ligand binding, synthesis, internalization, degradation, and recycling described in this model, these modeling experiments were expected to provide qualitative support for (or against) our postulated galectin lattice-mediated mechanism for 1,3,4-*O*-Bu_3ManNAc activity (**Figure 3** of the main manuscript). In particular, we utilized the model to guide our subsequent experiments wherein we anticipated that the galectin lattice mediated the impact of 1,3,4-*O*-Bu_3ManNAc on EGFR in a way beyond the previously reported direct dimerization mechanism. To illustrate the rationale behind use of the model, we tentatively ruled out the dimerization

mechanism. We gained additional support for discounting dimerization by using the model, which addressed EGFR dimerization indirectly by evaluating the k_{on} / k_{off} rates of EGF binding, and found (as described below) found these parameters did not reflect experimental results. By contrast, modeled simulations did align with expectations based on a galectin lattice mechanism --- reflected in internalization rates --- and moreover, also provided clues toward recycling mechanisms. Based on these results, we obtained (and report in the main manuscript) experimental evidence for perturbation of the galectin lattice in 1,3,4-*O*-Bu_3ManNAc-treated cells and also describe a shift away from clathrin-mediated endocytosis toward NCM internalization.

Procedures (i.e., Strategy for Model Implementation)

The model consisted of four differential equations with variables: free EGFR (Rs), internalized free EGFR (Ri), EGF-EGFR complex on surface (Cs), internalized EGF-EGFR complex (Ci). These equations account for the binding and release of EGF (L) from free EGFR (Rs), which are synthesized at rate S_r, at rates k_{on} and k_{off}, respectively. EGF-EGFR complexes on surface (Cs) are then internalized at rate k_{ec}, degraded at rate k_{xc}, and recycled at fraction f_{xc}. Free EGFR (Rs) is internalized at rate k_{er}, degraded at rate k_{xr}, and recycled at fraction f_{xr}. Internalization, k_e, is governed by rates k_{ec} and k_{er}. Degradation, k_x, is governed by rates k_{xc} and k_{xr}, while recycling, f_x, is governed by f_{xc} and f_{xr}.

The model was designed to simulate the conditions used in the western blots and EGF saturation binding experiments [7], which involved an overall incubation period of 48 h (with analog addition simulated through alterations in various rate constants) with serum starvation over the last 24 h, and finally 2 min exposure to 10 ng/mL of EGF. Rate constants then were varied incrementally to model the final values of total EGF-EGFR complexes (Cs+Ci), total available cell surface EGFR (Rs) and total EGFR (Rs+Ri+Cs+Ci). These values were then normalized against baseline values and then plotted against changes in rate constants normalized against baseline. The rate constants manipulated were k_{on}, k_{off}, and collectively, k_e, k_x.

Results and Discussion

Overview and Basic Considerations. The modeling simulations were based on the equations shown in **Figure S2B**, which covered parameters relevant to experimental endpoints described in our previously published work that showed that 1,3,4-*O*-Bu_3ManNAc treatment led to decreased levels of p-EGFR (Cs+Ci) and no significant change in total amount of EGFR (Rs+Ri+Cs+Ci) for a short time period (i.e., for 2 min) after addition of EGF [7]; in addition the model accounted for the lower levels of surface-localized EGFR (Rs) present on the cells immediately before the addition of EGF as shown in **Figure 3** of the main manuscript. In each set of simulations (as illustrated in **Supplemental Figure S3**) the predicted levels of total EGFR are shown in solid black and phosphorylated EGFR are shown in hollow circles in the upper graph, while predicted changes in surface expression of EGFR are depicted on the lower graph.

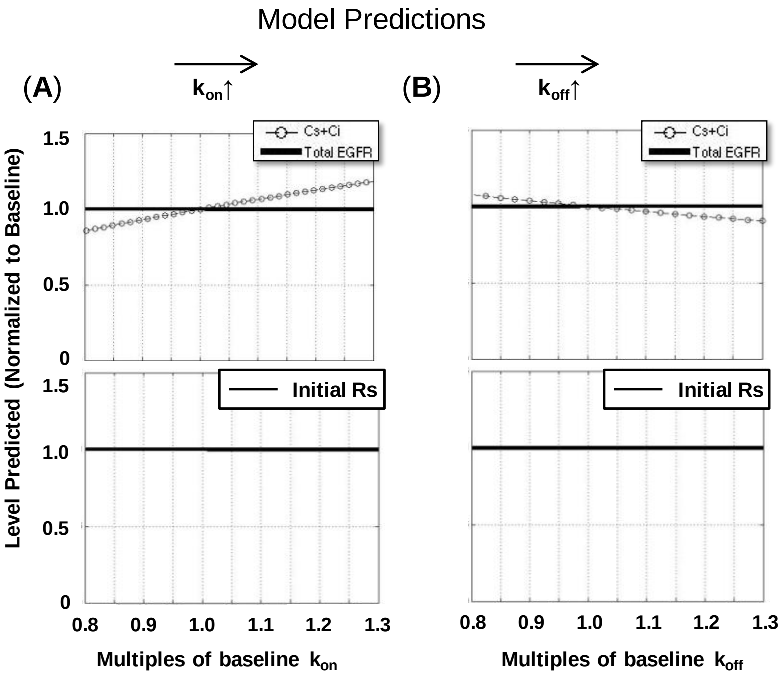

Supplemental Figure S3. **Results for simulated changes in k_{on} and k_{off} rates.**

Illustrative Results: Evaluation of k_{on} and k_{off}. Results illustrative of the usefulness of the mathematical simulations are shown in **Supplemental Figure S3**. In panels (**A**) and (**B**) of this figure, respectively k_{on} and k_{off} rates were respectively increased from left to right. The simulation predicted that k_{on} values were correlated with EGFR phosphorylation without affecting overall protein levels (**Panel A, upper graph**), which was consistent with the impact of 1,3,4-*O*-Bu_3ManNAc on EGFR [7]. The k_{on} rate, however, did not have an impact on cell surface levels of EGFR (**Panel A, lower graph**), which was contrary to experimental data (as shown in **Figure 3** of the main manuscript). Similarly, when k_{off} values were varied (**Panel B**), the amount of surface-displayed EGFR was not altered, which again was inconsistent with our experimental findings. Because ligand binding is linked to the

dimerization status of EGFR [9-11], EGF binding kinetics were expected to differ between 1,3,4-*O*-Bu_3ManNAc-treated and control cells if EGFR dimerization had been perturbed by analog treatment. Because this expectation was not met, this experiment provided additional evidence that our metabolic flux-based method to achieve increased sialylation modulated EGFR activity through a different mechanism than the previously-reported changes to dimerization achieved through sialyltransferase over-expression.

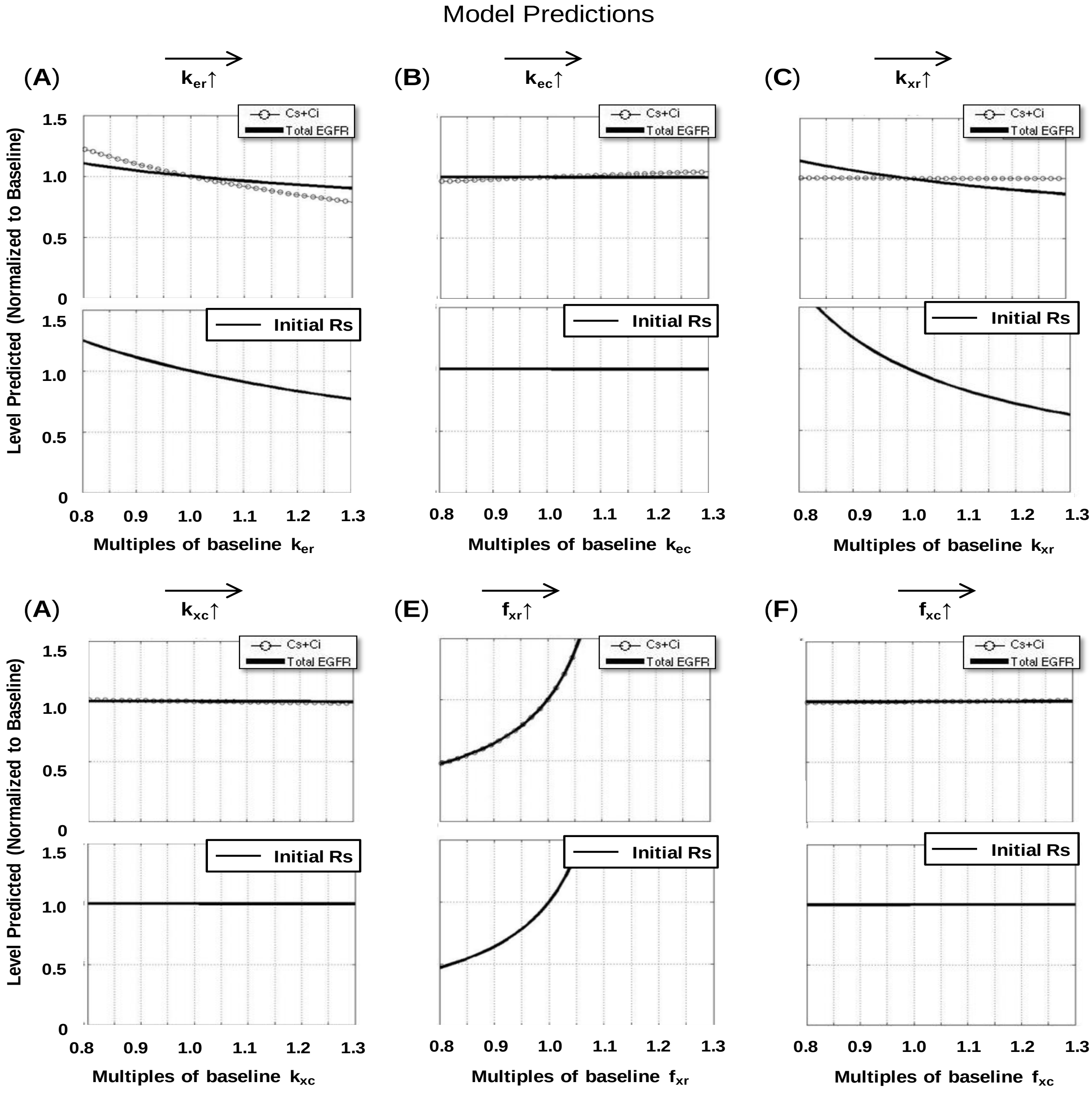

Supplement Figure S4. This figure shows an increased set of mathematical simulations that were conducted to ascertain if there were additional plausible mechanisms to account for the decreased levels of surface EGFR and cellular p-EGFR observed in 1,3,4-*O*-Bu_3ManNAc-treated SW1990 cells. Model output predictions, for changes in (A) k_{er}, (B) k_{ec},(C) k_{xr}, (D) k_{xc}, (E) f_{xr}, and (F) f_{xc}. In each case, predictions for changes in total EGFR are shown in solid black and phosphorylated EGFR are shown in hollow circles in the upper graph, while predicted changes in surface expression of EGFR are depicted in black on the lower graph. In addition, the parameters increase from left to right in all cases.

Expanded Set of Single-Parameter Simulations. Based on the outcome of k_{on} and k_{off} modeling simulations, which were not consistent with experimental data (as discussed above), we next systematically evaluated each of the other parameters in the model (as shown in **Supplemental Figure S2**). The results of these simulations are provided in **Supplemental Figure S4** and each panel is briefly discussed below:

Panel (A). Modeling of changes in internalization rate of available surface EGFR was not consistent with experimental data because while this endpoint did predict that an increase in internalization rate would lead to a decrease in EGFR phosphorylation and a decrease in surface bound EGFR, it also predicted that a similar decrease in overall EGFR levels would occur, which was not observed in the experimental data.

Panel (B). Modeling of changes in internalization rate of EGF-EGFR complexes was not consistent with experimental data because it does not predict significant changes from the baseline conditions.

Panel (C). Modeling of changes in recycling rate of unbound EGFR within endosomes was not consistent with experimental data because, while it did predict that an increase in recycling rate would lead to a decrease in available EGFR on the cell surface, it did not predict the decrease in EGFR phosphorylation that was previously observed.

Panel (D). Modeling of changes in recycling rate of EGF-EGFR complexes within endosomes was not consistent with experimental data because it did not predict significant changes from the baseline conditions.

Panel (E). Modeling of changes in recycling fraction of unbound EGFR within endosomes was not consistent with experimental data because while this endpoint did predict that a decrease in the recycling fraction would result in a decrease in EGFR phosphorylation and available surface bound EGFR (as observed) it also predicted a similar decrease in overall EGFR levels that was not observed experimentally.

Panel (F). Modeling of changes in recycling fraction of EGF-EGFR complexes within endosomes was not consistent with experimental data because no significant changes from the baseline conditions were predicted.

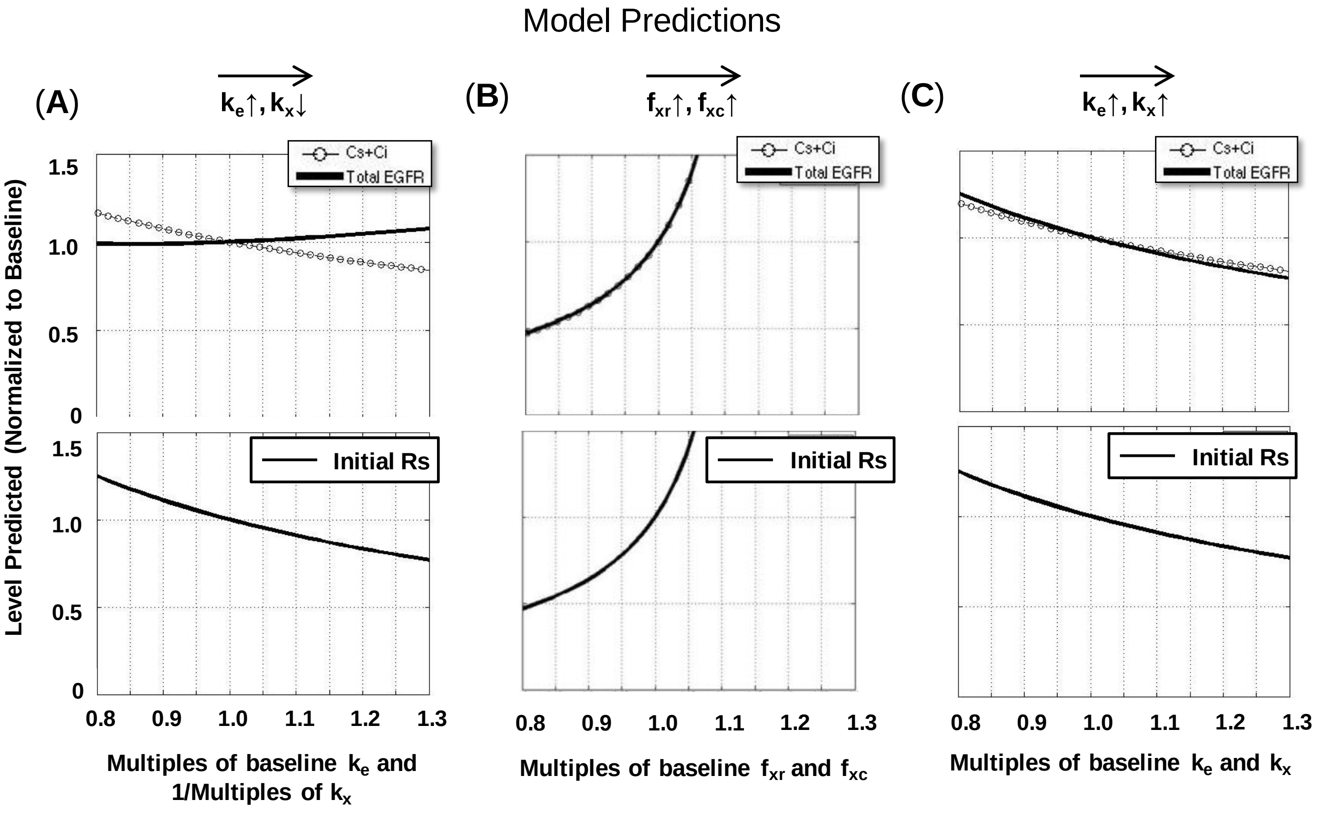

Supplemental Figure S5. Model output predictions for combined simulation of (A) k_e and $1/k_x$, (B) f_{xr} and, f_{xc}, and (C) k_e and k_x.

Pair-Wise Simulations. Based on the lack of insight from the single parameter simulations carried out as described above (and shown in **Supplemental Figures S3** & **S4**), we next systematically conducted simulations of all possible pair-wise combinations with three sets of results shown in **Supplemental Figure S5**.

The results shown in **Panel (A)** --- obtained by varying the simulated internalization and recycling rates --- proved to be the key simulation that provided a match between modeled and experimental results. Specifically, the model predicted (in the upper graph) that an increase in the internalization rate (k_e) along with a decrease in the recycling rate ($1/k_x$) would result in decreased EGFR phosphorylation accompanied by a slight increase in overall EGFR levels. Significantly, this set of variables also predicted (in the lower graph) the lower initial EGFR levels observed on the cell surface after treatment with 1,3,4-*O*-Bu_3ManNAc (i.e., as shown in **Figure 3** in the main manuscript) seen before the simulated addition of EGF. This simulation indicated that 1,3,4-*O*-Bu_3ManNAc increased the rate of EGFR internalization, consistent with the galectin lattice mechanism outlined in **Figure 4**. In addition, the decreased recycling rate ($1/k_x$) predicted was consistent with a shift towards non-clathrin mediated (NCM) endocytosis and away from clathrin mediated internalization because --- unlike clathrin-coated internalization --- NCM-internalized moieties are directed for degradation rather than recycling [12]. This insight led us to next investigate NCM endocytosis in more detail, which we experimentally confirmed in the main manuscript.

Another aspect of the pair-wise modeling simulations is that we evaluated all possible combinations. Two additional examples are shown in **Panels (B)** and **(C)** of **Supplemental Figure S5**:

Panel (B). Modeling of changes in recycling fractions of both unbound EGFR and EGF-EGFR complexes within endosomes was not consistent with experimental data because while it did predict that a decrease in both recycling fractions would result in a decrease in EGFR phosphorylation and available surface bound EGFR, it also predicted a similar decrease in overall EGFR levels that was not observed experimentally.

Panel (C). Modeling of changes in internalization and recycling rates of both available and bound EGFR was not consistent with experimental data because while it did predict that an increase in both the internalization and recycling rates would lead to an decrease in EGFR phosphorylation and a decrease in surface bound EGFR, it also predicted a similar decrease in overall EGFR levels that was not observed in the experimental data.

The remainder of the simulations are not shown or discussed herein because (i) the results did not match experimental data and (ii) the mechanistic implications of the combination are not readily apparent (i.e., any attempted explanation would of necessity be overly-speculative to be of any real value).

Flow cytometry histograms to support Figure 5 of the main manuscript

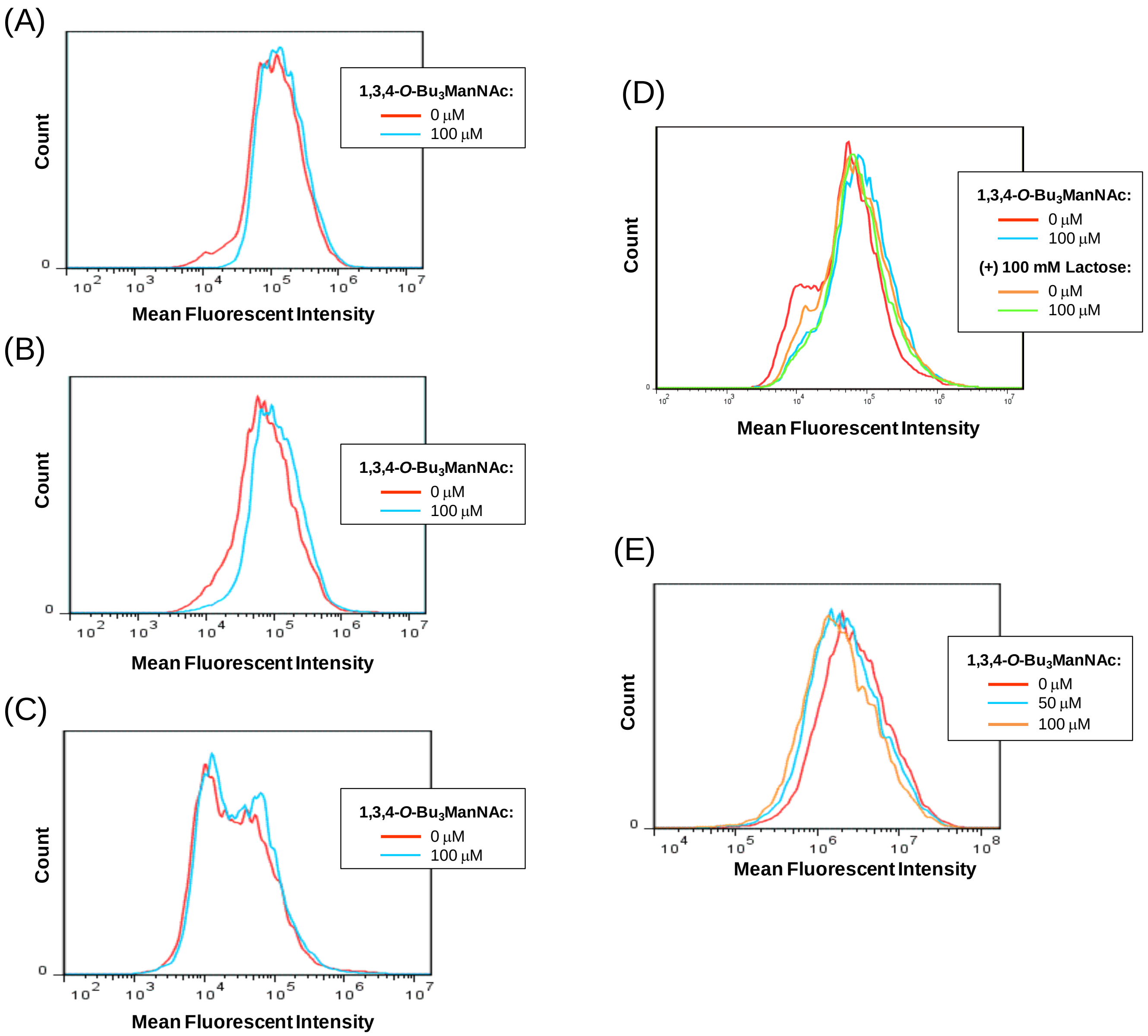

<u>Supplemental Figure 6</u>: EGFR internalization assays. Internalization assays both **(A)** before and **(B)** after acid wash showed that 1,3,4-*O*-Bu_3ManNAc treatment (100 μM) leads to a significant increase in EGF internalization. **(C)** Internalization measured after filipin pretreatment, however, was not significantly different between the control and treated samples. **(D)** Lactose pretreatment led to an increase in internalization in control cells comparable to the increase caused by 1,3,4-*O*-Bu_3ManNAc. **(E)** RCA lectin binding decreased significantly on treatment with 1,3,4-*O*-Bu_3ManNAc. At least 3 biological replicates were carried out for each experiment with data expressed as mean ± standard error mean (SEM). * indicates a p value of < 0.05.

REFERENCES:

1. Aich U, Campbell CT, Elmouelhi N, Weier CA, Sampathkumar SG, Choi SS and Yarema KJ. Regioisomeric SCFA attachment to hexosamines separates metabolic flux from cytotoxicity and MUC1 suppression. ACS Chem Biol. 2008; 3:230-240.

2. Wang Z, Du J, Che P-L, Meledeo MA and Yarema KJ. Hexosamine analogs: from metabolic glycoengineering to drug discovery. Curr Opin Chem Biol. 2009; 13:565-572.

3. Almaraz RT, Aich U, Khanna HS, Tan E, Bhattacharya R, Shah S and Yarema KJ. Metabolic oligosaccharide engineering with *N*-acyl functionalized ManNAc analogues: cytotoxicity, metabolic flux, and glycan-display considerations. Biotechnol Bioeng. 2012; 109:992-1006.

4. Almaraz RT, Tian Y, Bhattarcharya R, Tan E, Chen S-H, Dallas MR, Chen L, Zhang Z, Zhang H, Konstantopoulos K and Yarema KJ. Metabolic flux increases glycoprotein sialylation: implications for cell adhesion and cancer metastasis. Mol Cell Proteomics. 2012:10.1074/mcp.M1112.017558.

5. Shah P, Yang S, Sun S, Aiyetan P, Yarema KJ and Zhang H. Mass spectrometric analysis of sialylated glycans with use of solid-phase labeling of sialic acids. Anal Chem. 2013; 85:3606-3613.

6. Elmouelhi N, Aich U, Paruchuri VDP, Meledeo MA, Campbell CT, Wang JJ, Srinivas R, Khanna HS and Yarema KJ. Hexosamine template. A platform for modulating gene expression and for sugar-based drug discovery. J Med Chem. 2009; 52:2515–2530.

7. Mathew MP, Tan E, Saeui CT, Bovonratwet P, Liu L, Bhattacharya R and Yarema KJ. Metabolic glycoengineering sensitizes drug-resistant pancreatic cancer cells to tyrosine kinase inhibitors erlotinib and gefitinib. Bioorg Med Chem Lett. 2015; 25:1223-1227.

8. Hendriks BS, Opresko LK, Wiley HS and Lauffenburger D. Epidermal Growth Factor Receptor 2 (HER2) levels and locations. Quantitative analysis of HER2 overexpression effects. Cancer Res. 2003; 63:1130-1137.

9. Zhou M, Felder S, Rubinstein M, Hurwitz DR, Ullrich A, Lax I and Schlessinger J. Real-time measurements of kinetics of EGF binding to soluble EGF receptor monomers and dimers support the dimerization model for receptor activation. Biochemistry. 1993; 32:8193-8198.

10. Dawson JP, Berger MB, Lin C-C, Schlessinger J, Lemmon MA and Ferguson KM. Epidermal growth factor receptor dimerization and activation require ligand-induced conformational changes in the dimer interface. Mol Cell Biol. 2005; 25:7734-7742.

11. Macdonald-Obermann JL and Pike LJ. Different epidermal growth factor (EGF) receptor ligands show distinct kinetics and biased or partial agonism for homodimer and heterodimer formation. J Biol Chem. 2014; 289:26178-26188.

12. Sigismund S, Argenzio E, Tosoni D, Cavallaro E, Polo S and Di Fiore PP. Clathrin-mediated internalization is essential for sustained EGFR signaling but dispensable for degradation. Dev Cell. 2008; 15:209-219.
